# Documenting heritage language experience using questionnaires

**DOI:** 10.3389/fpsyg.2023.1131374

**Published:** 2023-05-23

**Authors:** Aleksandra Tomić, Yulia Rodina, Fatih Bayram, Cécile De Cat

**Affiliations:** ^1^Department of Language and Culture, UiT The Arctic University of Norway, Tromsø, Norway; ^2^School of Languages, Cultures and Societies, University of Leeds, Leeds, United Kingdom

**Keywords:** heritage language, individual differences, validation, language experience, questionnaire design, language entropy

## Abstract

**Introduction:**

There exists a great degree of variability in the documentation of multilingual experience across different instruments. The present paper contributes to the “methods turn” and individual differences focus in (heritage) bilingualism by proposing a comprehensive online questionnaire building on existing questionnaires and the experience of using them to document heritage bilingualism: the Heritage Language Experience (HeLEx) online questionnaire. HeLEx is validated against and contrasted to an extended version of the Language and Social Background Questionnaire designed for heritage speakers (HSs), LSBQ-H.

**Methods:**

We compare data elicited with both questionnaires in turn from a group of Turkish HSs (*n* = 174, mean age=32). Our validation focuses on traditional language background variables, including language exposure and use, language proficiency, language dominance, as well as a more novel measure of language entropy. The analyses are based on a subset of key questions from each questionnaire that capture language experience for up to five languages, four modalities, and five social contexts. In a subsequent set of analyses, we explore the impact of different types of response scales, response mechanisms, and manners of variable derivation on the informativity of the data they can provide, in terms of the scope, granularity and distributional properties of the derived measures.

**Results and Discussion:**

Our results show that both HeLEx and LSBQ-H are successful at detecting the important distributional patterns in the data and reveal a number of advantages of HeLEx. In the discussion, we consider the impact of methodological choices regarding question phrasing, visual format, response options, and response mechanisms. We emphasize that these choices are not trivial and can affect the derived measures and subsequent analyses on the impact of individual differences on language acquisition and processing.

## Introduction

In the last decades, it has become increasingly clear that the sociocultural and psycholinguistic experiences of multilingual individuals play a central role in shaping the diversity and variability of their linguistic and cognitive performance (cf., Green and Abutalebi, 2013; Abutalebi and Green, 2016; Titone and Tiv, 2022 for an overview). To understand the complexities of multilingualism, research has focused on identifying the key experience factors and their mediating role in characterizing multilingual language use, development, and cognition (e.g., Marian et al., 2007; Luk and Bialystok, 2013; Grosjean, 2015; Anderson et al., 2018; Bayram et al., 2019; Lloyd-Smith et al., 2020; Serratrice and De Cat, 2020; De Cat et al., 2023). Operationalizing multilingualism has become a line of research in itself, aiming to optimize the way we document and quantify the parameters of multilingual experiences. A number of instruments are available to inform this process, including ALDeQ (Alberta Language and Development Questionnaire, Paradis et al., 2010), ALEQ (Alberta Language Environment Questionnaire, Paradis, 2011), LEAP-Q (Language Experience and Proficiency Questionnaire, Marian et al., 2007), BiLEC (Bilingual Language Experience Calculator, Unsworth, 2013), BLP (Bilingual Language Profile, Birdsong et al., 2012; Gertken et al., 2014), BSWQ (Bilingual Switching Questionnaire, Rodriguez-Fornells et al., 2012), LSBQ (Language and Social Background Questionnaire, Luk and Bialystok, 2013; Anderson et al., 2018), LHQ 3.0 (Language History Questionnaire, Li et al., 2020), PaBiQ (Parental Bilingual Questionnaire, COST Action IS0804, 2011), and Q-BEx (Quantifying Bilingual Experience, De Cat et al., 2023). To some extent, these tools build on each other (e.g., PaBiQ builds on the ALDeQ and ALEQ, Q-BEx builds on the BiLEC, PaBiQ and ALEQ), reflecting advances in bilingualism research, both from a conceptual as well as a methodological point of view.

As highlighted in some recent review papers, there is a great degree of variability in the documentation of multilingual experience across different tools (Kašćelan et al., 2022; Rothman et al., 2023). While some are designed to estimate experience in early and late childhood (e.g., BiLEC, Q-BEx, and PaBiQ), others target adults (e.g., LSBQ). Crucially, the available instruments vary in how they capture the depth of bilingual experience since the specific components (exposure, domains of use, proficiency, dominance, etc.) are represented and measured with different levels of detail. There is variability in the set of communicative contexts considered, in the granularity of information about interlocutors and activities in each language, in the life periods documented, in whether language mixing is documented, and in whether attitudes are documented.

The recent “methods turn” in bilingualism research has brought to light issues of comparability of supposedly equivalent measures derived from different questionnaires (for in-depth discussion see Surrian and Luk, 2017; de Bruin, 2019; Kašćelan et al., 2022). This is due to variability in how the constructs of interest are operationalized (e.g., does the amount of exposure take into account the amount of time spent with different interlocutors?), but also in how the questions and response options are formulated. For instance, whether the amount of exposure to each language is recorded on a percentage scale or a Likert scale, whether the points on the Likert scale are labeled, and if so, whether they are labeled with numbers or qualifying terms (e.g., “rarely,” “most of the time”).

Beyond issues of documentation (i.e., what do we ask about and in what level of detail?), there is also variability in how the data is processed. For example, BiLEC, ALDEQ, PaBiQ, and Q-BEx propose specific algorithms to generate composite measures of children’s language experience. Quantity-focused measures include current exposure and use (adjusted according to the amount of time the child spends with different interlocutors or in different contexts) and cumulative exposure and use. Quality-focused measures include composite scores reflecting the diversity of the language experience in terms of interlocutors (e.g., the number of native or non-native speakers providing input, as well as different interlocutors with whom the child communicates exclusively in a given language) or contexts of use. For example, BLP offers an algorithm which automatically calculates the score for language dominance based on 19 questions distributed across four modules (language history, use, proficiency, and attitudes), which ranges from −218 to +218. The extreme values represent dominance in one vs. the other language and the middle values represent more balanced bilingualism.

Recently, language entropy has been proposed as a new measure of language experience (Gullifer and Titone, 2020), inspired by previous work on language mode and social diversity of language use (Grosjean, 2001, 2015). The concept originates in Shannon’s (1948) theory which defines entropy as a measure of information content and uncertainty. Entropy was previously used in psycholinguistics (Hale, 2003; del Prado Martín et al., 2004; Levy, 2008) and neurocognition (Gullifer et al., 2018). In the context of multilingualism, language entropy is derived from estimates of exposure to different languages in different social/communicative contexts. It can be interpreted as a measure of social diversity, indexing the level of non-uniformity in the daily usage of two or more languages across contexts: high entropy scores are indicative of high language diversity in a given communicative context and therefore low language certainty, while low entropy scores are indicative of low diversity and comparatively higher language certainty. For instance, a context in which the individual regularly interacts in their multiple languages in a balanced manner would have high entropy. Another context in which the individual predominantly interacts in a single language would have low entropy. Bilinguals with high language entropy experience a greater number of language states across their communicative contexts than bilinguals with low entropy. Therefore, they may experience less certainty about which language they will be exposed to in a given context. Contrary to the measure of language dominance, language entropy is not indicative of *which* language takes precedence in a given context. It therefore is a valuable addition to other, established measures.

The LSBQ focuses on bilinguals’ language usage patterns, in different contexts and with different individuals in daily life. The LSBQ goes further than most tools, as it aims to derive a *unique* composite score estimating the degree of bilingualism (of young adults). The composite score it generates operationalizes an important dimension in recent theorizing about the bilingualism effect, namely the role of interactional context in determining the degree of bilingualism an individual possesses. It is also possible to calculate multiple composite scores based on the distribution of how a bilingual uses each language in different domains of life (home vs. work vs. social contexts). The composite score reflects the extent of proficiency and use of languages other than the societal language, both within and outside of the home. Based on this, bilinguals can be assigned into groups defined along a monolingual-to-bilingual continuum: a composite score of less than −3.13 would categorize one as monolingual, while having a score above 1.23 would be regarded as being bilingual. The most recent version of the LSBQ (Anderson et al., 2018) follows the footsteps of its predecessor version (Luk and Bialystok, 2013) but bears similarities to other existing tools such as the LEAP-Q (Marian et al., 2007) and the LHQ 2.0 (Li et al., 2006, 2014). This latest version of LSBQ has been validated across a large sample size (*n* = 408) of young adults using exploratory factor analysis (EFA).

This brief review shows that the documentation and operationalization of bi-/multilingualism is an incremental endeavor, reflecting research development in terms of scope and in terms of methods. New and more precise measures become necessary (e.g., various aspects of language mixing, language entropy), and existing measures are revisited to enhance their reliability.

The current paper fits within this incremental tradition, by presenting a new questionnaire to inform heritage bilingualism research: the Heritage Language Experience questionnaire (HeLEx). Our initial intention had been to “simply” adapt LSBQ for online data collection, as it is a validated, established, fairly exhaustive, and one of the most commonly used questionnaires to qualify and quantify heritage bilingual experience. However, we found ourselves adding and modifying questions in an attempt to augment and optimize LSBQ to meet our research needs: we added several components focusing on language attitudes, code-switching attitudes and behavior, decided to separate the use of and exposure to languages, and to ask about quantity and quality of language experience in absolute terms. To minimize the data wrangling required to derive language entropy measures and to facilitate the derivation of other composite measures, questions are asked in relation to the same five social contexts of interaction throughout the questionnaire. The formulation of questions and response scales is informed by the psychometric literature (Dillman et al., 2014, 2016). Furthermore, we attempted to remain as neutral as possible in the question text and response options, to reduce any potential emotional discomfort associated with the often minoritized or stigmatized status of heritage language, within the immigrant-origin community and the larger society. For example, code-switching is a frequent, yet often stigmatized language behavior in heritage language communities and beyond. When probing the frequency of use and exposure to code-switching, the question preamble explains that research shows that it is a frequent and normal behavior in many multilingual communities. When probing code-switching attitudes of our participants and other people in their lives, the negative attitude option was carefully chosen not to attach any strong or objective negative value to code-switching, resulting in the following option list: “It should be avoided,” “I do not have an opinion,” “It’s okay,” “I do not know.”

This study is an empirical evaluation of these modifications to LSBQ. Our first objective is to validate HeLEx against (an augmented version of) the LSBQ (i.e., LSBQ-H), by comparing data elicited with both questionnaires from the same group of HSs (i.e., Turkish HSs living in Germany), first with LSBQ-H and then with HeLEx several months to a year later. The validation focuses on traditional background variables, such as language exposure and use, language proficiency, language dominance, as well as a more novel measure of language entropy. Comparing questionnaire data for the same participants allows us to shed light on the impact of different types of response scales, both on the distribution of the raw data, and on the distribution of derived measures. Our second objective is to explore the informativity of each questionnaire (HeLEx vs. LSBQ-H) in terms of the scope and granularity of the derived measures.

## Methodology

### LSBQ-H: an extended version of LSBQ for heritage speakers

The original LSBQ comprises three sections: (1) social background/demographic information, including age, education, country of birth, immigration, and parents’ education as a proxy of SES; (2) information about language background, i.e., questions about which language(s) the participant can understand and/or speak, age of acquisition, etc., as well as questions about self-rated proficiency for speaking, understanding, reading and writing the indicated languages; and (3) information about community language use, including language use in different life stages (infancy, preschool age, primary school age, and high school age), language use and code-switching in specific contexts (with different interlocutors), in different situations (home, school, work, and religious activities), and for different activities (reading, social media, watching TV and browsing the internet).

As a first step, an expanded version of the LSBQ was created (Bayram, 2021, unpublished) to optimize it for the documentation of heritage bilingualism, by adding and expanding questions to document the following aspects of HS’ experience in more detail: (i) HL language training and formal education in HL; (ii) changes in language experience over the lifetime (documenting changes for up to three languages, across several periods); (iii) language profile of partners or cohabitants; (iv) parental language, immigration history and education in each language; (v) visits to the country of HL origin and size of HL community in the current society; (vi) different patterns of code-switching; and (vii) community language attitudes.

The rest of the questionnaire was implemented as in the original^1^ with a few minor exceptions: (i) for the frequency of use by modality question, “Of the time you spend engaged in each of the following activities, how much of that time is carried out in [language]?,” response options changed from “None,” “Little,” “Some,” “Most,” “All” to “None,” “Very little,” “50–50,” “Most,” “All”; (ii) the response options for the HL use proportion, out of use of HL and another language, in different contexts were changed to acknowledge that the participants likely speak more than two languages, so the proportion of HL use is now estimated out of use of all languages. The LSBQ-H was administered online, using the Gorilla questionnaire builder, as a part of a larger study on Turkish as a HL in Germany. In the transfer to the online version of the questionnaire, the LSBQ-H attempted to replicate the LSBQ-on-paper visual format.

### The Heritage Language Experience questionnaire

In creating the Heritage Language Experience questionnaire (HeLEx), three main principles were adopted: (i) expand coverage to capture the multi-faceted HL experience, (ii) adopt recommendations from the psychometric literature to optimize response scales, (iii) keep frames of reference as constant as possible, e.g., inquire about the same language contexts within and across questions.

The HeLEx questionnaire was developed to capture the individual language experience of heritage bilinguals primarily. Therefore, it includes most variables which could potentially affect HL acquisition and processing while minimizing questionnaire completion time. The questionnaire can also be used by immersed bilinguals in general. This is useful since the language experience of the first-generation of immigrants providing input to heritage bilinguals could be captured using the same questionnaire.

The full questionnaire contains the following modules:

- Demographics,
- Visits to HL country,
- Proficiency in five languages in four modalities (speaking, listening, reading, and writing),
- Language experience for five languages in four modalities and in tech-related activities,
- Diversity and quantity of HL and societal language (SL) experience in five different social contexts (Home, External Family, Work/School, Leisure, Community),
- Proportion of HL speaking and listening in five social contexts,
- Code-switching types and frequency in five social contexts,
- Attitudes (both personal and community) on code-switching in five social contexts,
- Historical use of HL and SL in self-defined periods,
- HL literacy training,
- Personal language attitudes,
- Community language attitudes.

**Figure 1:**
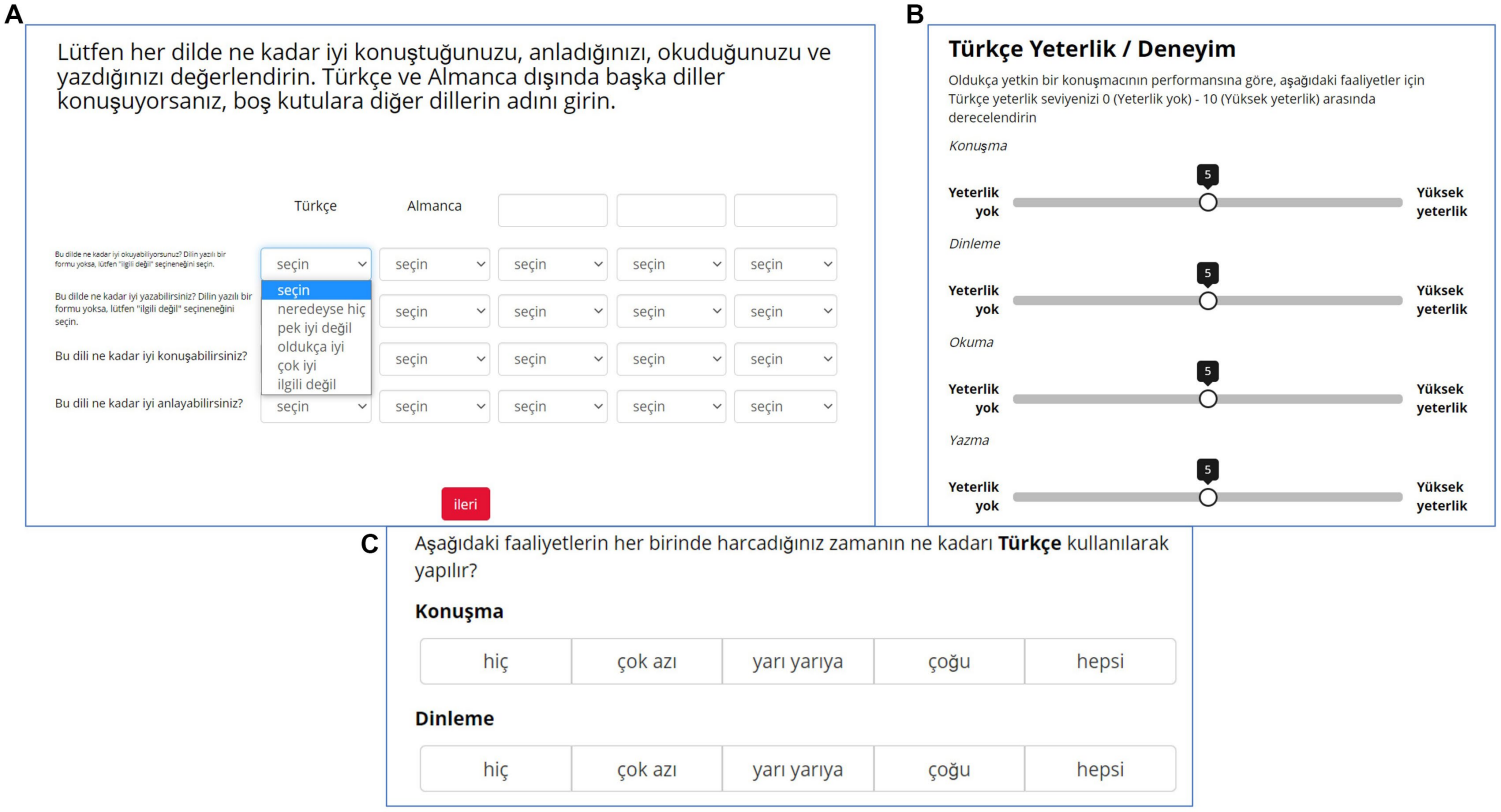
(**A**; top left). An example of a matrix visual format question with dropdown menu response mechanism from HeLEx, probing the proficiency in HL (Turkish), SL (German), and 3 additional languages (columns), in speaking, understanding, reading, and writing (rows). (**B**; top right). Single column visual format example with sliders as response mechanism from LSBQ-H, probing the proficiency in Turkish in 4 modalities. **(C)** Example of an LSBQ-H question using clicking on a button response mechanism probing the relative frequency of HL use.

Contrary to LSBQ, the questionnaire does not assume the existence of particular interlocutors (e.g., parents, siblings), and does not require making any assumption about household composition or any other context. We introduced these indirectly grouped and consistent frames of reference for contexts across questions to reduce thinking time when filling the questionnaire. It also allows the straightforward combination of information across questions during data wrangling. For example, to probe the diversity and quality of the HL input, HeLEx asks, for each context, how many interlocutors the participant spends any time within a typical week, how many of them have good HL proficiency, and how many of them are dominant in HL. Helping respondents maintain the same context/inquiry focus in mind while responding was achieved by using matrices (tables) for the majority of questions. The matrix questions target sets of contexts and/or sets of languages, with each field in the matrix usually containing a dropdown menu with response options (see Figure 1 for an illustration). This would be difficult to achieve in a paper version, as the response options could not fit on a page.

Another affordance of the online interface is the use of sliders for answers expressing proportions (e.g., HL use) or level of agreement (e.g., attitudes). This was intended to be more visually intuitive by avoiding overt quantization, hence reducing cognitive burden. The potentially more fine-grained responses might also capture more accurately the individual reality of HL experience.

As in LSBQ (Dunning et al., 2004; Anderson et al., 2018), we allowed some level of redundancy in some questions probing key concepts for triangulation (i.e., questionnaire-internal validation). For instance, to probe language experience in different social contexts, HeLEx uses both response scales based on natural metrics (number of days, number of hours) and proportion-based response scales (e.g., sliders ranging from “HL only” to “other languages only”). In contrast, the LSBQ primarily uses ratios (e.g., HL vs. other most used/proficient languages). HeLEx uses both a question in absolute time terms (“How many days per week do you meet speakers of [HL]/[SL],^2^ at least some of them?,” “How many hours do you typically spend together with them?”) as well as relative time indicators of HL and other language use using slider scales ranging from “HL only” to “other languages only” spoken and heard in the five contexts.

Since it targets heritage bilinguals, the questionnaire implementation in Gorilla is optimized to be translated and offered in both the HL and SL.^3^ The Gorilla HeLEx questionnaire is freely available for use at the Gorilla Open Materials page.^4^ It is also developed for use in Qualtrics, to ensure wide availability. The Gorilla implementation of the HeLEx questionnaire is accompanied by an R script (available at the OSF repository https://osf.io/mkjax) which provides automatized numerical transformation of textual responses and calculation of derived variables, including, among others, language entropy (using the R package languageEntropy, release v1.0.1c, Gullifer and Titone, 2018). This set of derived variables captures the multi-faceted HL experience.

Each dropdown menu in HeLEx began with the prompt “select” and many menus included “does not apply” option at the end, to easily identify non-responses (as dropdown menu widgets in Gorilla Task Builder cannot be set to require a response). Instructions at the beginning of the questionnaire stated that participants should always select an appropriate dropdown option even if they believe the question does not apply to them, and that questions left on “select” will be considered not responded to. Questions with the “select” as the response were quantized, i.e., numerically transformed, to NaNs (“not a number”).

Despite many additional questions, the average completion time for HeLEx was 10.5 min (sd = 7.02) for the 227 Turkish-German bilinguals who took the questionnaire, and around 11 min (sd = 7.61) for the 174 participants whose data was analyzed, as opposed to the average LSBQ-H completion time of 5.45 min for the 174 participants included in the analysis. We believe the affordances of the online implementation and keeping fewer contexts consistent across the questionnaire contributed to the relatively short completion time for HeLEx considering the number of questions, but we cannot exclude factors such as population characteristics (uniform or extreme experience, clear intuitions on language use and attitudes) and previous engagement with language experience questionnaires. In any case, the focus on individual variables in language processing means that questionnaires now must be on equal footing with tasks in terms of importance and therefore time commitment for participants (within reason).

### HeLEx validation methods

Our aim is to validate HeLEx by comparing its derived measures with those from the LSBQ-H, using data collected from the exact same population of heritage speakers using each questionnaire in turn. Both questionnaires are available in spreadsheet format from the OSF repository at https://osf.io/mkjax.

The first set of analyses aims to ascertain whether the two questionnaires reliably capture the same reality, insofar as the distribution of the resulting measures is sufficiently similar. The objective threshold we use for “sufficient similarity” is the absence of statistically significant questionnaire effect on the distribution of key variables. These key variables are those frequently used as predictors in the bi−/multilingualism literature: language exposure and use, language proficiency, language dominance, and language entropy. The second set of analyses explores the informativity of each questionnaire, in terms of scope and granularity of the derived measures. Both sets of analyses consider methodological choices, in terms of question phrasing, visual format, response options, and response mechanism, and their effect on measures. We then discuss the implications of our findings.

#### Participants

Two hundred and twenty-seven (227) Turkish-German HSs took both LSBQ-H and HeLEx. The LSBQ-H data for 13 participants who took HeLEx was not available, so they were excluded. Out of these 214 participants, 40 participants were excluded due to not having data on language use for most social contexts. This was likely due to a glitch, as the same sequence of questions was missing across participants. The mean age of the participants included in the analysis (66 men, 108 women) was 32.08 (sd = 4.67, range 23–47). The vast majority (168) was born in Germany, whereas six moved to Germany at or before the age of 3. Out of 116 participants who reported living with someone, only two lived with partners who did not speak Turkish. In most other cases, the partner spoke Turkish as their first or native language. When asked by LSBQ-H on the size of the HL-speaking community, most of the participants (144) reported having an intermediate to massive community. Twenty-one participants reported having reading and/or writing lessons in Turkish in mainstream German public schools and six reported having had additional reading and/or writing lessons in Turkish. Thus, it seems that our participants belong to a thriving, connected HS community, with many opportunities for HL acquisition, use, and maintenance. It is likely that snowball sampling and self-selection further ensured that the sample includes participants with high use and proficiency of HL.

#### Procedure

The questionnaires were administered in Turkish. The English versions used in this paper consist in (i) the original English version of HeLEx and (ii) a back-translation into English of the Turkish LSBQ-H. LSBQ-H was administered first, within a larger study, and HeLEx around half a year to a year later.

#### Questions used to derive the measures of interest

The subset of questions used in this validation analysis are those required to derive the variables of interest, as explained above. These questions were asked differently by the two questionnaires, in terms of content of the questions, response elicitation mechanism, and visual format. The main visual format of the selected questions was either a Matrix or a Single column. Unlike the single column question (Figure 1B), the matrix question presents the same questions, usually arranged in the rows of the first column, for several different languages or contexts, with one column reserved for each language or context (Figure 1A).

The response mechanism could either be selecting an option from a dropdown menu (Figure 1A), moving the slider tip to the desired point on scale (Figure 1B), or clicking on a button (Figure 1C). Different visual configurations could be combined with different response mechanisms.

Another point of difference is the number of options, whether the options were presented verbally and numerically (or both), and whether the response options included absolute or relative quantities (usually in relation to the other language or languages). The main characteristics of the questions selected to measure the concepts of interest are summarized in Table 3.

Tables 1, 2, 4, 5 below summarize and contrast the questions across questionnaires.

**Table 1:** HeLEx and LSBQ-H version of the question(s) on the proficiency in up to five languages in four modalities.

	HeLEx	LSBQ-H
Instructions	Please rate how well you speak, understand, read and write in each language. Enter the name of other languages in the boxes, if you speak other languages than [HL] and [SL].	Rate your [HL] proficiency for the following activities, based on a highly competent speaker’s performance level from 0 (No qualification) to 10 (Higher proficiency) for the following activities. Rate your proficiency level in your most familiar/used language outside of [HL] for the following activities, based on a highly competent speaker’s performance level from 0 (No qualification) to 10 (Higher proficiency) for the following activities.
Languages probed	[HL], [SL], Language 1, Language 2, Language 3	[HL], most familiar/used language outside of [HL]
Question structure	Matrix: languages across columns; modalities across rows	Two separate questions for each language, not following each other, with modalities vertically ordered
	How well do you speak it?	Speaking
	How well do you understand it?	Listening
	How well do you read in it?	Reading
	How well do you write in it? If the language does not have a written form, please select “not relevant.”	Writing
Response mechanism	Dropdown menu	Slider scale
Response options	hardly at all, not very well, pretty well, very well, does not apply	No proficiency (0) – high proficiency (10)
Response options, quantized	1, 2, 3, 4, 0	0, 1, 2, 3, 4, 5, 6, 7, 8, 9, 10

**Table 2:** HeLEx and LSBQ-H versions of the question on frequency of use of up to five languages in four modalities.

	HeLEx	LSBQ
Instructions	For all languages you use, rate how frequently you use them. Enter additional languages you might speak in addition to [HL] and [SL].	How much of the time you spend doing the following activities is spent using [HL]? How much of the time you spend doing the following activities is spent using other most proficient language?
Languages probed	[HL], [SL], Language 1, Language 2, Language 3	[HL], other most proficient language
Question structure	Matrix: languages across columns; modalities across rows	Two separate questions for each language, not following each other, with modalities vertically listed
	How often do you hear it?	Listening
	How often do you speak it?	Speaking
	How often do you read it?	Reading
	How often do you write in it?	Writing
	How often do you do any computer/technology-related activities in each language? E.g., TV, radio, music, films, websites, games, apps.	This is probed in a different series of questions in LSBQ.
Response mechanism	Dropdown menus	Horizontally ordered buttons
Response options	1. (Almost) never, 2. A few times per year, 3. Once a month, 4. Once a week, 5. A few times per week, 6. Once per day; most days, 7. Several times per day; most days	Never, very little, 50–50,^1^ most, all
Response options quantized	1, 2, 3, 4, 5, 6, 7	1, 2, 3, 4, 5

1 The original response option list from LSBQ is *None, Little, Some, Most, All*.

**Table 3:** The comparison of HeLEx and LSBQ-H questions used in the analysis and their characteristics.

Concept	Questionnaire	Visual format	Response mechanism
SL and HL proficiency	HeLEx Proficiency	Matrix	Dropdown menus
	LSBQ-H Proficiency	Single column	Sliders
SL and HL experience in diff. modalities	HeLEx Experience in diff. modalities	Matrix	Dropdown menus
	LSBQ-H Experience in diff. modalities	Single column	Buttons
SL and HL experience in different social contexts	HeLEx Proportion of HL use in social contexts	Matrix	Sliders
	LSBQ-H Proportion of HL use in social contexts	Single column	Buttons
	HeLEx Diversity and quantity of input/exposure to HL and SL	Matrix	Dropdown menus

**Table 4:** HeLEx and LSBQ-H questions on the proportion of HL experience in social contexts.

Questionnaire	HeLEx	LSBQ-H	
Instructions/questions	Think of all interactions in a typical week. For each context consider face to face and online communication. How much do you speak in each language in each context? How much do you hear each language in each context? The more you speak one language the closer you should put the slider to it. If the slider is in the middle that means you speak [HL] and [SL] in equal amounts. Put the slider all the way to the left if you only speak [HL] and no [SL] in this context. Put the slider to the left but not all the way if you mainly speak [HL] but sometimes use [SL] in this context.	Please indicate which languages you speak generally with the following people. (Please leave relevant columns empty if they do not apply)	Please indicate which language(s) you use generally in the following situations. (Please leave relevant columns empty if they do not apply)
Visual format	Matrix: 2 columns (modalities) by 5 rows (contexts)	Single column	
Modalities	Speaking, Hearing	Use (not explicitly defined)	
Contexts	Family in the household; family outside the household; work or school; local community (shops organizations etc.); leisure (hanging out with friends or roommates, hobbies)	Mother–Father; Siblings; Grandpa(s)-Grandma(s); Other relatives; Friends; Partner; Housemates; Neighbors	Home; School; Work; Social activities (spending time with friends, watching movies, etc.); Religious activities; Out-of-school activities (hobbies, sports, volunteer activities, computer games, etc.); Shopping/Restaurant/Other commercial activities; Health services/Government-public institutions/Banks
Response mechanism	Slider	Buttons (horizontally ordered)	
Response options	0–100 in steps of 1 (slider tip initially presented at 50 mark)	Only [HL], Mostly [HL], Half [HL] half other language(s),^1^ Mostly other language(s), Other language(s) only	
Response options, quantized	0–1 (in steps of 0.1)	1, 0.75, 0.5, 0.25, 0	

1 The original LSBQ response option list is All [language], Mostly [language], Half English half other language, Mostly the other language, Only the other language.

**Table 5:** The specifics of the HeLEx matrix questions on the quantity, quality, and diversity of HL and SL experience.

Introduction	Think of all the people you interact with in [HL]/[SL] in a typical week in different contexts including face-to-face and online interaction.
Visual format	Matrix: contexts in each column, question in each row
Contexts	family in the household; family outside the household; work or school; local community (shops, organizations, etc.); leisure (hanging out with friends, roommates, hobbies)
Response mechanism	Dropdown menu

Questions	How many people do you use [HL]/[SL] with?	How many days per week are you with these people (at least some of them)?	On a typical day when you are with these people, how many hours do you spend together in total?	How many of these people speak [HL]/[SL] very well?	For how many of these people is [HL]/[SL] their best language?
Response options	0, 1, 2, 3, 4, 5, 6–8, 9–11, 12–14, 15–17, 18–20, more than 20	not every week, 1, 2, 3, 4, 5, 6, 7	less than 1, 1, 2, 3, 4, 5, 6, 7, 8, more than 8	0, 1, 2, 3, 4, 5, 6–8, 9–11, 12–14, 15–17, 18–20, more than 20	0, 1, 2, 3, 4, 5, 6–8, 9–11, 12–14, 15–17, 18–20, more than 20
Response options, quantized	0, 1, 2, 3, 4, 5, 7, 10, 13, 16, 19, 22	0.5, 1, 2, 3, 4, 5, 6, 7	0.25, 1, 2, 3, 4, 5, 6, 7, 8, 9	0, 1, 2, 3, 4, 5, 7, 10, 13, 16, 19, 22	0, 1, 2, 3, 4, 5, 7, 10, 13, 16, 19, 22

##### Overall experience in each language in different modalities

HeLEx and LSBQ-H versions of the question on frequency of use of five languages in four modalities. As shown in Table 2.

##### Proportion of HL use by social context

The specifics of the HeLEx matrix questions on the quantity, quality, and diversity of HL and SL experience. As shown in Table 4. HeLEx and LSBQ-H version of the question(s) on the proficiency in five languages in four modalities. As shown in Table 5.

##### Proficiency in each language in 4 modalities

The comparison of HeLEx and LSBQ-H questions used in the analysis and their characteristics. As shown in Table 1.

### Derived measures

#### HL experience and proficiency in four different modalities

Calculating the scores for HL experience and proficiency in four modalities required minimal derivation, i.e., simple numerical transformation, presented in question summary tables, in case the response options were presented verbally.

#### Proportion of HL use in different social contexts

##### HeLEx data selection and preparation

HeLEx probes the following five contexts:

- Home (including whoever lives in the household)
- External family (family outside the household)
- Work or school
- Local community (shops, organizations, restaurants etc.)
- Leisure (hanging out with friends, roommates, hobbies).

To derive the proportion of HL use in different contexts, we used two questions, detailed in Tables 4, 5. HL use was probed in two ways to compare the effects of different response mechanisms (slider vs. dropdown) and different ways of calculating proportions of HL use (directly from responses in the case of sliders, and by deriving proportion of time of HL exposure from absolute time responses on the quantity of use of HL and SL). The slider question on HL use readily provides the proportion of HL speaking and listening out of all language use in each context, with minimal derivation (0–100 to 0–1.0 transformation). The proportion of the (potential) HL exposure was also calculated from the questions on the diversity and quantity of language exposure which probe the time spent with HL and SL speakers in a typical week in absolute terms: number of days spent with HL speakers and SL speakers in each context and the amount of time in hours spent with them in total on a typical day. We calculated the proportion of time that participants spent with HL speakers out of all time spent with speakers of any language in a typical week in the following way:

1. For each context in each language (HL, SL), we multiplied the total number of daily hours spent with speakers of that language by the number of days per week spent with these speakers in each context to obtain interaction hours (In the case there were no HL or SL speakers met in a context the interaction time in that particular language and context was set to 0.). For each language, we then calculated the sum of interaction time in hours across contexts.
2. We calculated the total amount of hours of HL and SL interaction per week, by summing the time spent with HL and SL speakers across contexts.^5^
3. We then calculated the proportions of HL exposure per week by dividing the hours of the exposure to HL by the total hours spent with any speakers per week.

##### LSBQ-H data selection and preparation

LSBQ-H documents language experience through a mix of by-person and by-context questions (Table 4). While this provides precision and granularity, it is difficult to group the measures into larger meaningful contexts. The proportions of HL vs. SL use in the Home and Work/School contexts in HeLEx are comparable to the following LSBQ-H context questions:

- Home HL experience = quantized proportion of HL use in the home out of other languages (0, 0.25, 0.5, 0.75, 1.0).
- Work or School HL experience = the highest value out of Work HL use proportion and School HL use proportion, or simply the one that was responded to.

The External Family, Community, and Leisure contexts were more difficult to reconstruct using the collected LSBQ-H responses. When this question is asked in HeLEx for each context, e.g., Leisure, it is understood that participants do not have to have each of the subcontexts represented in their lives equally, e.g., for Leisure, hanging out with friends, roommates, hobbies. Because the wording of the question and the entire questionnaire is geared towards the HL experience, they are simply giving an answer on the use of HL vs. SL in each context as a whole, and likely choosing subcontexts with the highest HL experience representation. In eliciting responses this way, we maximally avoid researcher-imposed definitions of each context.

To group LSBQ-H responses on the use of HL vs. other language(s) with individual persons and different situations into a smaller number of more meaningful subcontexts, we could take the mean of several responses on the use with specific speakers/in specific situations corresponding to a particular subcontext. For example, we could average the response to the question on Social activities (spending time with friends, watching movies, etc.), Religious activities, and Out-of-school/work activities (hobbies, sports, volunteer activities, computer games, etc.), to get at the HL use in the Leisure context. Nevertheless, Religious activities might not be a significant part of each participant’s experience during leisure and might not be taken into account while responding to the same question (Leisure) in HeLEx. Whatever such participants respond to the question on HL experience during religious activities, unless they skip it, it will distort the participant’s social context reality when calculating the HL experience mean. We thus took a conservative approach and reconstructed the HeLEx contexts in LSBQ-H calculations using the least controversial subcontexts and transformations, and not penalizing for non-responses for subcontexts.

We attempted to reconstruct the External Family HL experience, a monolithic context defined as “family outside the home” in HeLEx, by taking the mean of HL experience with grandparents and other relatives from LSBQ-H, the family members most likely to live outside the home. Nevertheless, this is a reach in conclusion, since we cannot for certain know which family members cohabitate with participants and which do not. Also, the data for this context behaved differently to others in early plots, suggesting that the approximation was likely not successful. Therefore, we present the comparisons of measures for External Family within HeLEx only and not between HeLEx and LSBQ-H.

The Local Community HL experience was reconstructed by using the mean HL experience with neighbors and while shopping (key subcontexts the majority of participants should have):

- Local community HL experience = mean of HL use ratio with neighbors and in the local community, i.e., while shopping, visiting restaurants, and other commercial activities.

The Leisure HL experience was reconstructed by using the mean of HL experience during extracurricular and social activities:

- Leisure HL experience = mean of HL use ratio during extracurricular activities, i.e., hobbies, sports, volunteering, playing games, and social activities, i.e., hanging out with friends.^6^

#### Dominance

We derived two Dominance variables from the data reviewed so far: one based on experience in each language in four modalities, and the other based on proficiency in each language in four modalities. Dominance in each modality was operationalized as a ratio, by dividing the relevant HL measure by the relevant SL measure for each modality.^7^ The overall dominance for both measures was calculated as the mean of the HL over SL score ratios for the four modalities (i.e., speaking, listening, reading, writing). For the ratio calculations, a value of around 1 indicates that the participant is balanced overall in terms of HL vs. SL (exposure or use), when all modalities are considered. A value above 1 indicates dominance in HL in terms of experience or proficiency. It is important to note that the overall dominance scores for proficiency and experience may hide variation across modality-specific dominance scores.

#### Language entropy

We used the data on Proportion of HL use in different social contexts derived from HeLEx and LSBQ-H responses (section *Derived measures: Language entropy*) and the R package languageEntropy (release v1.0.1c, Gullifer and Titone, 2018) to calculate language entropy for the following contexts:

- Home: Family in the household
- Work or School
- Local community (shops, organizations, restaurants etc.)
- Leisure (hanging out with friends, roommates, hobbies)

For HeLEx, we exploit two types of context-specific language experience questions: (i) a question probing the proportional use of HL and other language(s) using sliders (Table 4), and (ii) questions probing experience of SL and HL separately (Table 5). In both cases, the questions are asked about the following contexts: Home, External Family, Work/School, Leisure, Community. For LSBQ-H, the five macro-social contexts were reconstructed from ratios of HL use with individual speakers/in specific situation, as detailed in the section *Derived measures: Proportion of HL use in different social contexts*. The same proportions of use were used to calculate language entropy.

#### Accounting for the actual proportion of time spent in each context

Traditionally, language experience questionnaires such as LHQ and LSBQ have measured proportions of exposure to the HL language (or use) with a specific individual or in a specific context out of the total use or compared to the SL. However, participants may be spending different amounts of time in each context/with each individual. For example, someone might report that they use the HL 45% of the time at home, whereas another respondent may report 90% use in that context. Imagine the first respondent actually spends 8 h per day at home, whereas the second respondent spends only four. This needs to be taken into account to calculate the total amount of HL experience contributed by the home context (which is equivalent for respondents 1 and 2).

To account for this, we developed weights based on the proportion of time spent with speakers of either language in each context out of the total time spent with speakers during a typical week (Diversity and Quantity of HL and SL input question). This can only be done with responses from HeLEx, as LSBQ or LSBQ-H do not provide absolute time response options for language use.

To derive these weights, we first calculated the total amount of time spent with any speakers. We multiplied the typical number of total hours participants spend with HL speakers on a typical day in each context by the number of days per week they meet with these speakers in each context. We then repeated the process for the SL speakers. To get the number of hours spent with speakers of either language per week, we summed the number of hours spent with HL and SL speakers across contexts (this does assume that the HL and SL speakers are separate speakers). We then calculated the proportion of time spent in each context out of the total time spent with anyone per week, adding up to 1. These context weights were then multiplied with the proportions of the HL vs. SL use in each context.

For unweighted scores, the total HL exposure would be approximated by the average of HL exposure across contexts (i.e., the sum of HL proportions per context divided by the number of contexts). For weighted estimates, the total HL exposure approximation would be the across-context sum of HL exposure in each context multiplied by the weight for that context. Therefore, we compare averages of unweighted scores for HL use proportion across contexts and *sums* of weighted scores across contexts.

#### Diversity of HL interlocutors

HeLEx provides information about the number of interlocutors in each context, the number and proportion of speakers with good proficiency in HL in each context, and the number and proportion of speakers who are dominant in HL in each context (see Table 5). These measures are useful to approximate the quality and diversity or variation in the input. For each context, the proportion of interlocutors with good HL proficiency is calculated by dividing the number of such interlocutors by the total HL interlocutors in that context. The same procedure was used to derive the proportion of interlocutors who are dominant in HL in each context, as well as overall proportions across contexts. Importantly, the calculations include a data-validation check ensuring that the number of HL-dominant or HL-proficient interlocutors in each context does not exceed the total number of HL interlocutors in the context reported by the respondent (this resulted in a negligible data loss for this sample).

### Statistical analysis methods

We employed linear regression models for all relevant statistical comparisons (probing main effects of questionnaire, type of calculation, contexts, modalities, and interactions between them), using lmer() (lme4 package, version 1.1-31, Bates et al., 2015) or lm() function (stats package, version 3.6.2) in R (version 4.2.2, R Core Team, 2022). Random intercepts for participants were included where supported by the data. In the linear regression models, we applied dummy contrast coding to the questionnaire variable, where HeLEx was the reference level. In the case of Context and Modality variables, which had four or more levels, we applied deviation coding, where the estimate for each context or modality level was made in reference to the mean of means of HeLEx values across contexts or modalities. The variables were recoded and models were rerun where necessary to obtain the estimates for the contexts or modalities initially coded to-1 (e.g., for Work and Writing). The model outputs can be found in the Supplementary material.

## Results

### Validation

To assess the similarity across the two questionnaires, for each variable of interest, (i) we illustrate the distribution of the variable as per each questionnaire, (ii) we fit a regression model to ascertain if there is a statistically significant questionnaire, context, modality, or manner of calculation effect for the variable in question.

#### Overall experience in HL in different modalities

The relevant HeLEx and LSBQ-H questions probing the frequency of use of languages in four modalities (reading, writing, speaking, and listening/understanding) are shown in Table 2. LSBQ-H elicits a ratio/percentage of the use of HL vs. “the other most proficient language” which is established earlier in the questionnaire and is usually the SL. HeLEx probes the use of HL and SL, as well as three additional languages in absolute frequency terms (cf., response options). HeLEx also includes a sub-question on the use of the languages in tech-related activities, as they are often multimodal and therefore eschew quantification when probed by using the terms such as writing/reading/listening, etc. LSBQ-H contains a section on the use of HL relative to SL during separate tech-related activities, e.g., social media, TV, etc.

**Figure 2:**
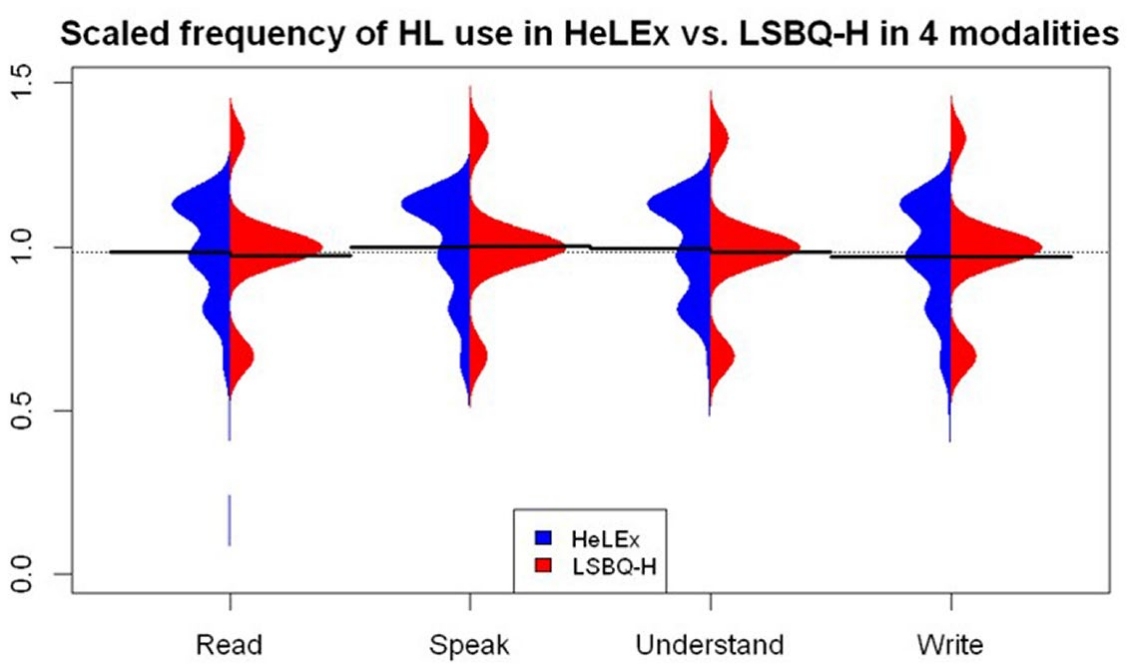
Scaled scores for the frequency of HL use (by modality) from the HeLEx and LSBQ-H questions listed in Table 2.

The responses were gathered by the two questionnaires using different Likert scales: different number of points on each scale (7-point scale for HeLEx and 5-point scale for LSBQ-H), and different labels for each scale. To facilitate the comparability of responses, we quantized scores and then scaled them: each score was divided by the standard deviation of the frequency scores distribution from the relevant questionnaire, using the scale() base R function. This is a common standardization method, often performed on independent variables with differing scales before entering them into regression models as predictors. A scaled score of 1 denotes that the unscaled score is equal to the standard deviation of the corresponding distribution. The value of standard deviation will of course be directly dependent on the scale. Nevertheless, transforming the scores in terms of the number of standard deviations for each distribution makes the two scores directly comparable. The scaled distributions are shown in Figure 2.

The spread of the distributions seen in Figure 2 is determined by the distribution of actual responses (rather than the range of the original response scales). As the top and bottom ends of the scale were not used by LSBQ-H respondents, the scores are distributed across three scores, corresponding to the red peaks in Figure 2. By contrast, the HeLEx scores feature a more continuous distribution. We entered the main effects of the Questionnaire and Modality, as well as their interactions, into the linear mixed effects model as potential predictors of HL experience scores, with random intercepts for participants.

The results of the linear regression model (Supplementary Table S1, Supplementary material) indicate no statistically significant difference between the estimates obtained from the two questionnaires in each modality when controlling for different scales. Compared with the mean of means of exposure/use across modalities and questionnaires (i.e., the model intercept) in HeLEx, writing in the HL is significantly less frequent, and speaking in the HL is significantly more frequent. There was no significant interaction between the questionnaire and modality. We can conclude that the two questionnaires provide a similar distribution of HL experience in different modalities, albeit with a different level of granularity.

#### Proportion of HL use across social contexts

**Figure 3:**
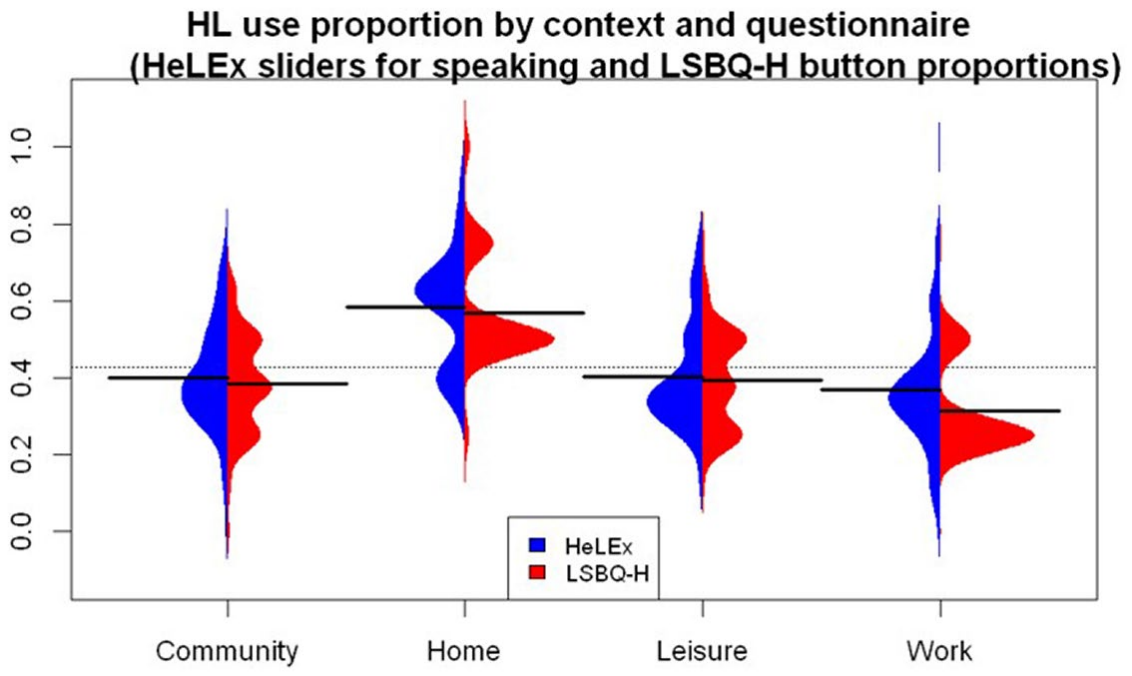
The distribution of the use of HL (speaking modality) out of all languages in each context, derived from HeLEx and LSBQ-H (for reasons mentioned in the section *Derived measures: Proportion of HL use in different social contexts*, we do not compare the External Family context across questionnaires).

For comparing the two questionnaires on the measure of the proportion of HL use overall per social context, the question on the proportion of HL speaking out of all languages elicited with sliders from HeLEx (0–100 scale, Table 4) was chosen as the closest equivalent to the LSBQ-H question on the proportion of HL *use* elicited with horizontally ordered buttons (5-point scale, Table 4). Visually (Figure 3), it seems that the proportion of HL use was highest in the Home context, and lowest in the Work or School context. LSBQ-H seems to return lower estimates of HL use, compared with HeLEx. We entered the main effects of Questionnaire and Context, as well as their interactions, into the linear regression model as potential predictors of HL use proportions.

The linear regression model (Supplementary Table S2) confirmed that the LSBQ-H-derived estimates of the proportion of HL use are significantly lower than those of HeLEx, albeit with a small estimate value. This is likely due to this specific choosing the LSBQ/LSBQ-H “Mostly [HL]” response option when the reality of their experience was between the “Only [HL],” quantized as 1, and “Mostly [HL],” quantized as 0.75. In other words, the participants an option higher than 0.75 and lower than 1, but chose the lower 0.75 to avoid the beginning point of the scale.

In terms of cross-context comparison, the HL use seems to be significantly lower in the Community, Leisure, and Work or School contexts, whereas it is significantly higher in the Home context compared to the mean of means of the HL use proportion across all contexts as measured by HeLEx. There was also a significant interaction between the questionnaire (LSBQ-H) and context (Work or School), such that LSBQ-H provided even lower estimates for the Work or School context.

#### Proficiency in the HL in four modalities

**Figure 4:**
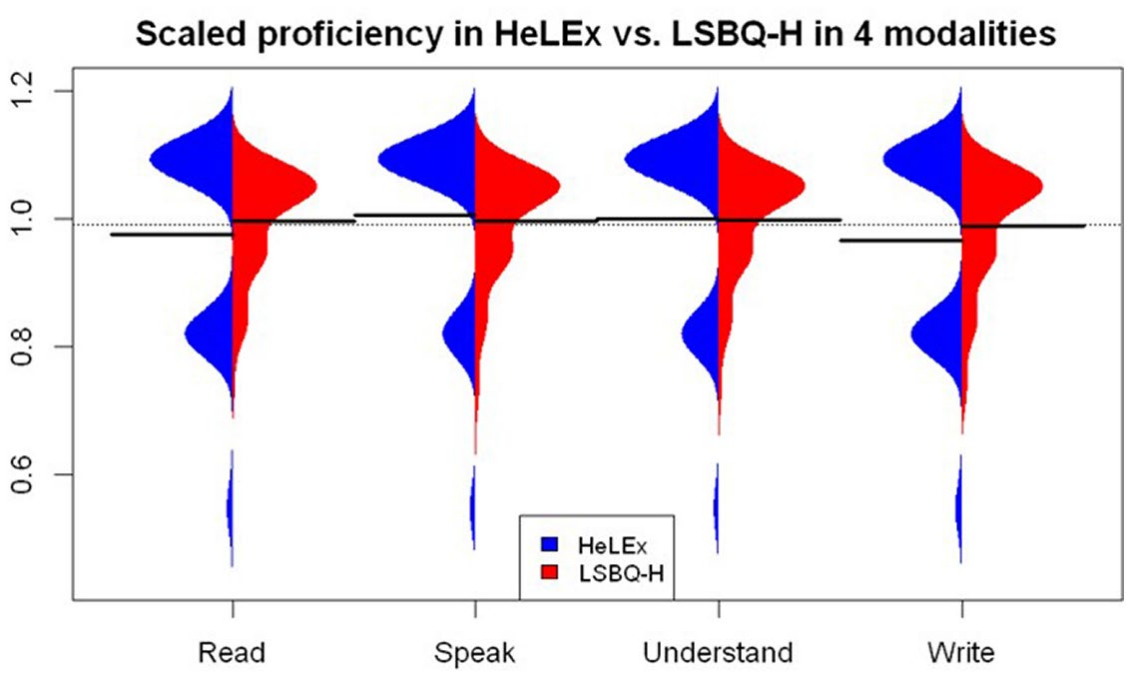
Response distribution for HL proficiency by modality in HeLEx and LSBQ-H, scaled.

For proficiency, the LSBQ-H response scale is more granular (i.e., 11 points) than that of HeLEx (4 points). The LSBQ-H also presents the options numerically, with minimal use of evaluative language, unlike HeLEx. Importantly, the LSBQ-H online adaptation includes the slider as the response mechanism, as the most appropriate equivalent of a visual scale with a pronounced mid mark in the paper LSBQ version. The numeric, more granular LSBQ-H slider scale resulted in a more exponential distribution with a high concentration of top-of-the-scale responses compared to the evaluative dropdown menu, as seen in Figure 4. We entered the main effects of the Questionnaire and Modality (i.e., speaking, reading, understanding, and writing), as well as their interactions, into the linear mixed effects model as potential predictors of HL proficiency scores, with random intercepts for participants.

The results of the linear mixed effects model reveal that, despite the visual differences in the distribution, no statistically significant difference was found between the LSBQ-H-and HeLEx-derived measures of proficiency when controlling for the differences in scale, illustrated in Supplementary Table S3. Expectedly, the sample reports a higher proficiency in HL Speaking and lower proficiency in Writing compared to the mean of means of HL proficiency across all modalities as recorded by HeLEx. There was no significant interaction between the questionnaire and modality.

### Dominance

#### Experience-based dominance scores

**Figure 5:**
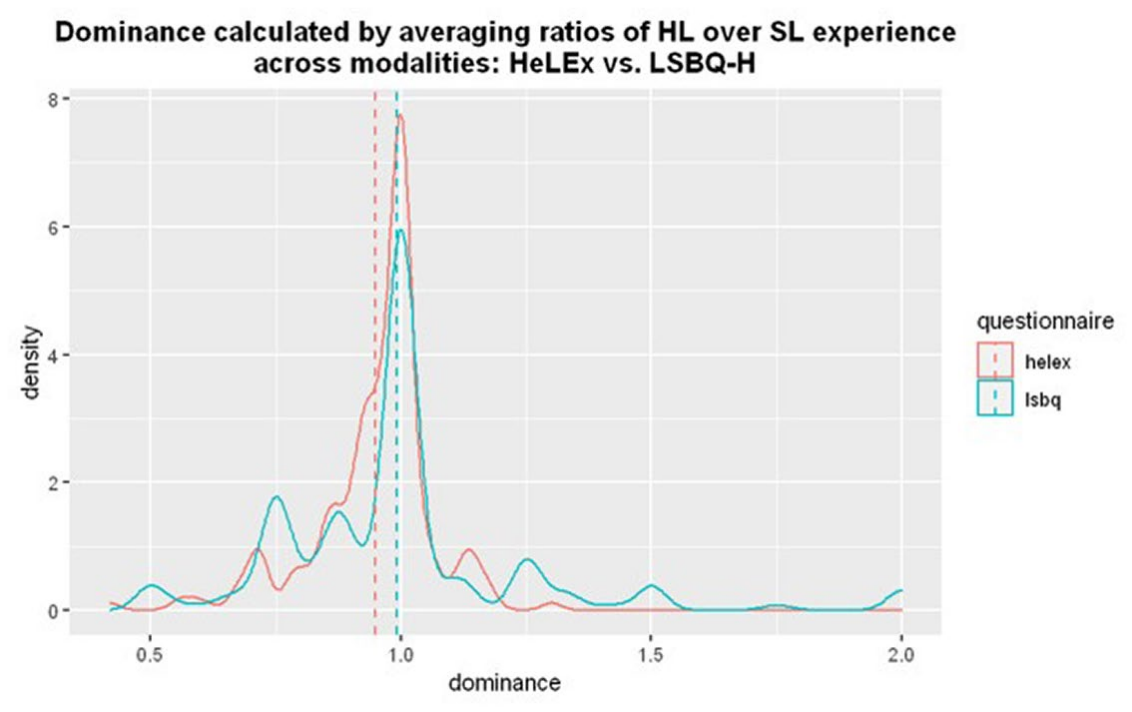
The density plot for the distribution of dominance calculated by averaging ratios of HL over SL experience in different modalities from HeLEx (red) and LSBQ-H (blue). The density on y axis represents a kernel density estimate, a smoothed version of frequency on y axis in a regular histogram.

Figure 5 shows that most people in the sample are balanced bilinguals. The measure calculated with LSBQ-H responses is possibly more discriminatory (with less clustering around 1), but a more diverse population would be needed to assess this. We fitted a linear regression model to assess whether the estimates of Experience-Based Dominance were predicted by the questionnaire used. There was a small but significant effect of the questionnaire, such that LSBQ-H Experience-based HL dominance estimates seem to be higher than those of HeLEx (Supplementary Table S4).

#### Proficiency-based dominance scores

**Figure 6:**
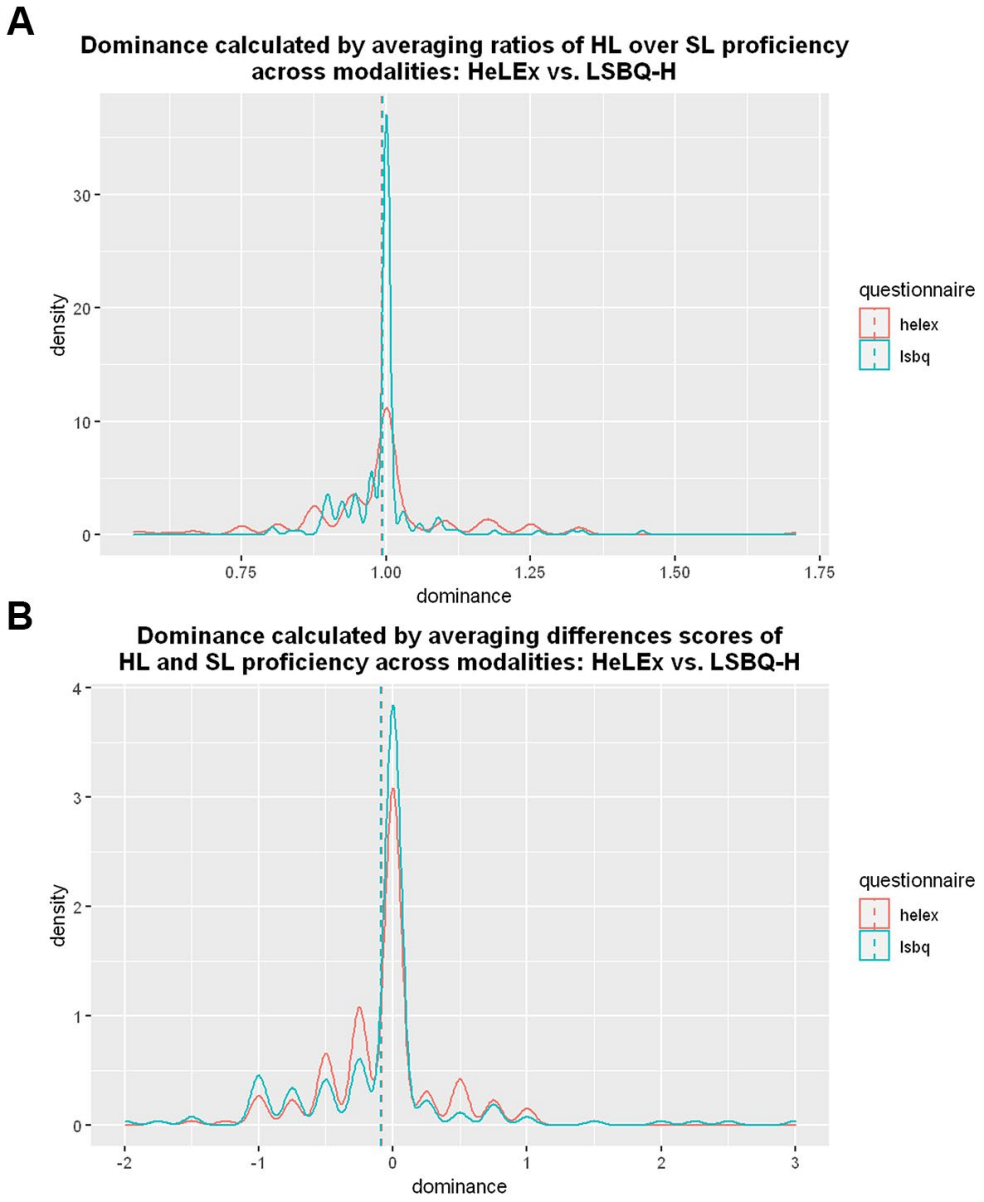
**(A)** The density plot for the distribution of dominance calculated with ratios of HL vs. SL proficiency in different modalities from HeLEx (red) and LSBQ-H (blue). **(B)** The density plot of the dominance-by-proficiency scores calculated by averaging difference scores between HL and SL proficiency measures across 4 modalities, from HeLEx responses (red) and LSBQ-H responses (blue).

The proficiency-based dominance distribution using ratios is smoother for the HeLEx measures than the LSBQ-H measures, despite LSBQ-H providing more response options (Figure 6A). There is a strong concentration of balanced proficiency values (around 1) for LSBQ-H results. We entered the main effects of Questionnaire into the linear mixed effects model as potential predictors of HL proficiency-based dominance scores calculated using ratios.

The results of a linear regression model in Supplementary Table S5 suggest that there is no significant difference between the proficiency-based ratio dominance scores derived from HeLEx and LSBQ-H, despite visual differences in distribution. The distributions are affected by differences in the original response scales. A 1-point difference is smaller on a 10-point scale than on a 4-point scale: a relatively “balanced” participant with high proficiency in both languages might have a 9:10 dominance ratio (=0.9) as per LSBQ-H and a 3:4 ratio (=0.75) as per HeLEx. The resultant “bunching” of balanced scores is therefore more marked for LSBQ-H than for HeLEx, as seen in Figure 6A. As an alternative method, we also derived a difference score for dominance-by-proficiency, by subtracting SL proficiency from HL proficiency. For this type of calculation, the value of 0 (no difference) would indicate balanced proficiency.

Figure 6B shows that the difference scores provide much more similar distributions of dominance-by-proficiency between the two questionnaires. Here, despite the differences in the original response scales, the resulting scores are more similar across questionnaires. The same relatively “balanced” proficient participant (as discussed above) would have a dominance difference score of –1 (i.e., 9 for HL proficiency minus 10 for SL proficiency) as per LSBQ-H, and a dominance difference score of –1 (i.e., 3 for HL proficiency minus 4 for SL proficiency) as per HeLEx. In a less balanced population sample, the distributions of difference scores would differ more across questionnaires (as the maximum difference score is 9 for LSBQ-H vs. 3 for HeLEx). Both methods nonetheless concur in showing that the vast majority of the participants in our sample are balanced bilinguals, with a slight leaning to SL dominance: the mean dominance score from both questionnaires is to the left of the balance score (i.e., 1 for ratio calculations and 0 for difference score calculations) in each plot.

#### Language entropy by context

**Figure 7:**
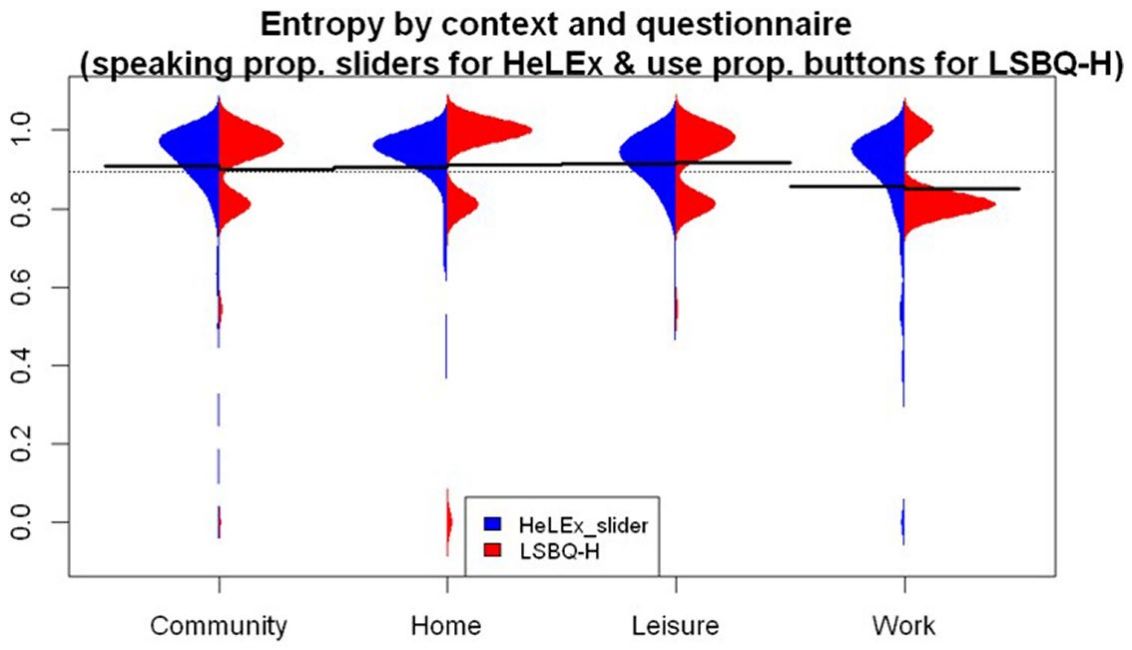
The distribution of language entropy calculated using proportions of HL speaking in social contexts from HeLEx slider questions (blue) and proportions of HL use in LSBQ-H in reconstructed contexts.

Figure 7 plots the language entropy measures by context for each questionnaire. Both questionnaires seem to return a slightly higher language entropy for the Home and Leisure contexts. The lowest entropy is found in the Work or School context. We entered the main effects of the Questionnaire and Context as well as their interactions into the linear regression model as potential predictors of language entropy scores.

The model summary (Supplementary Table S6) shows that there is no significant difference in entropy estimates between the two questionnaires. The entropy in the Work or School context was significantly lower than the mean of entropies across all contexts as recorded by HeLEx. Focusing on this particular context reveals marked differences in the distribution of scores across questionnaires, in spite of similar means (HeLEx mean = 0.856, sd = 0.191; LSBQ-H mean = 0.852, sd = 0.104). The data points are concentrated around the highest value for HeLEx, indicating high entropy, whereas the majority of responses are at a lower mark for LSBQ-H. The HeLEx values are overall more distributed due to the slider scale providing more options than the options for the ratio of HL vs. SL use in the LSBQ-H questions where entropy scores only included four possible values.

### Interim summary

We considered a range of (mainly standard) measures of HL experience and compared the measures derived from the LSBQ-H data with those derived from the HeLEx data. The measures included HL Experience (across modalities and across contexts), HL proficiency (across modalities), language dominance (based on experience and based on proficiency), and language entropy. Despite some small differences, the results were generally similar across questionnaires, concluding the validation of HeLEx. We now turn to the affordances of HeLEx and discuss their methodological implications.

### Informativity effects: code-switching and attitudes

HeLEx is characterized by additional informativity compared to LSBQ-H as it includes an extended code-switching (CS) module, an extended section on personal and societal attitudes towards HL, an extensive section on the personal and societal attitudes to CS in five contexts, questions on the number of speakers of HL and SL in each context and their HL proficiency, among others.

The CS module of HeLEx probes the frequency of personal CS use as well as CS exposure in the five social contexts for two directions of code-switching (HL to SL and SL to HL) and for three structural types (one word, two to three words, intersentential CS). When asked how often they use or are exposed to a specific type of CS in each context, the participants had the following general options: “(almost) never, in one or two conversations per week, in one or two conversations per day, in (almost) every conversation, I do not know.”

In the battery of questions on personal and societal attitudes to the HL use and knowledge, participants use sliders (0–100) to indicate how much they agree with specific statements. Personal attitudes statements include, among others, “I identify myself as [a national of the HL matrix country],” “It is important that my children learn [HL] to a high degree,” “It is important to me to speak and understand [HL] like speakers who live in [the HL matrix country],” “I am satisfied with my current overall ability in [HL].” Societal attitudes statements include “I am worried that speaking my home language is not welcome/tolerated in the wider society,” “There is sufficient support from the government and society for maintaining my home language,” “I feel external pressure to speak in the dominant language of the society, either by colleagues, friends, etc.” The personal attitudes questions are mostly co-opted from the Bilingual Language Profile (Gertken et al., 2014). These questions can be grouped into several scores (e.g., the importance of HL for self-identification, satisfaction with and perceived importance of HL knowledge, etc.), or they can be averaged to create an index of positive attitudes to HL.

Additionally, HeLEx affords the opportunity for triangulation in relation to the documentation of HL experience, as some aspects are probed by two similar sets of questions. We investigate discrepancies between different types of response scales, and their impact on derived measures. We also illustrate the additional informativity of HeLEx with the results for the questions on the input diversity (number of speakers of each language in each context) and the HL input quality.

### Estimates of the proportion of HL experience: natural metrics vs. estimated proportions

**Figure 8:**
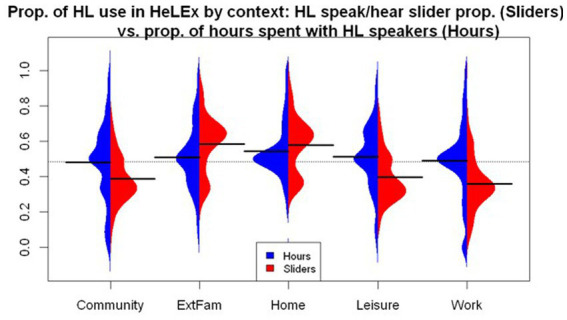
The distribution of proportion of HL use across contexts based on HeLEx data, comparing hour-based responses with slider-based responses.

The proportion of HL use can be calculated in two ways using HeLEx data. One method, using natural metrics data, divides the hours spent with HL speakers in a particular context (in a typical week) by all hours spent with anyone in that context (in a typical week). The other method, using estimated measures, is based on the proportion of HL vs. other languages (elicited via sliders), averaging the values for speaking and hearing.^8^ The resulting distributions are shown in Figure 8.

Both methods reveal similar trends across contexts. The proportion of HL use in the Home is the highest, whereas it is the lowest in the Community and Work or School contexts. However, the proportions calculated with slider responses seem to exhibit more marked by-context differences than the proportions obtained from hour-based responses. We entered the main effects of the Questionnaire and the Manner of the HL Use Proportion Calculation, as well as their interactions, into the linear mixed effects model as potential predictors of HL proficiency scores with random intercepts for participants. The Manner of Calculation variable had Hours as the reference value.

The results of the linear mixed effects regression model (Supplementary Table S7) suggest that the HL proportion calculated using slider responses is significantly lower overall. In terms of contexts, Community has a significantly lower HL use proportion estimate, whereas Home has a higher estimate, compared to the mean of means of HL proportion across contexts for the hour-based calculation. The significant interaction of Context and Manner of calculation suggests that slider-derived estimates are higher for the Home and External Family contexts and lower for the Work or School, Community, and Leisure contexts.

The slider responses might provide more categorical estimates: they further amplify the HL proportion trends for the Home and Community context. They also might be more reliable, as they give direct estimates of the proportion of language experience in each context and should also reflect the proportion HL use during language mixing. By contrast, the hour-based data is a derived measure with more steps, and it only reflects the time spent with HL interlocutors in each context (irrespective of the actual HL use proportion with these people, in case they are bilinguals).

#### Deriving language entropy from interaction hours vs. slider data

**Figure 9:**
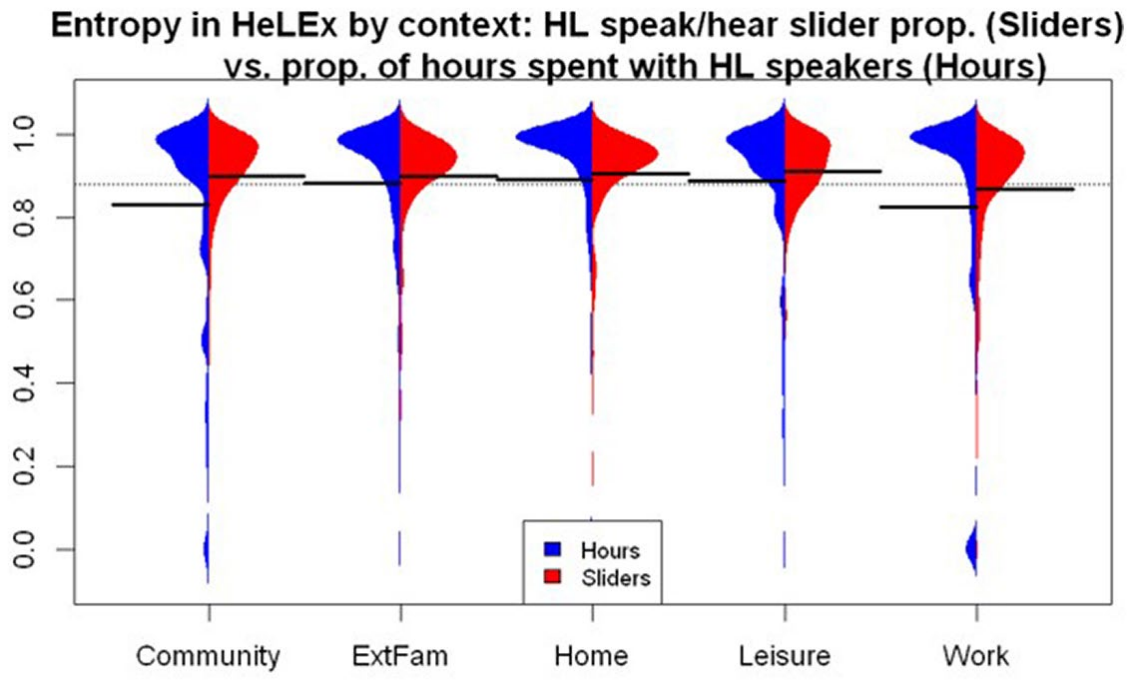
The distribution of language entropy by context calculated from HeLEx slider responses (red) and the question on the quantity of time spent with HL and SL speakers (blue).

We derived language entropy scores from the two estimates of HL experience we have just compared, yielding the distributions shown in Figure 9.

We entered the main effects of the Manner of Calculation and Context, as well as their interactions, into the linear mixed effects model as potential predictors of entropy scores, with random intercepts for participants. The Manner of Calculation variable had Hours as the reference value.

The linear mixed effects model summary in Supplementary Table S8 shows that, compared to the mean of means of entropy for all contexts as calculated using hours, language entropy is significantly lower in the Community and Work or School contexts and higher in the Home and Leisure contexts. The significant main effect of the Manner of Entropy Calculation suggests that entropy calculated using slider responses is significantly higher, possibly due in part to the inclusion of potential non-responses (sliders left on 0.5 translating to high entropy). The interaction between the Manner of Calculation and context suggests that the estimates for Community entropy are significantly higher for the slider-derived calculation.

#### Considering the time spent in each context

**Figure 10:**
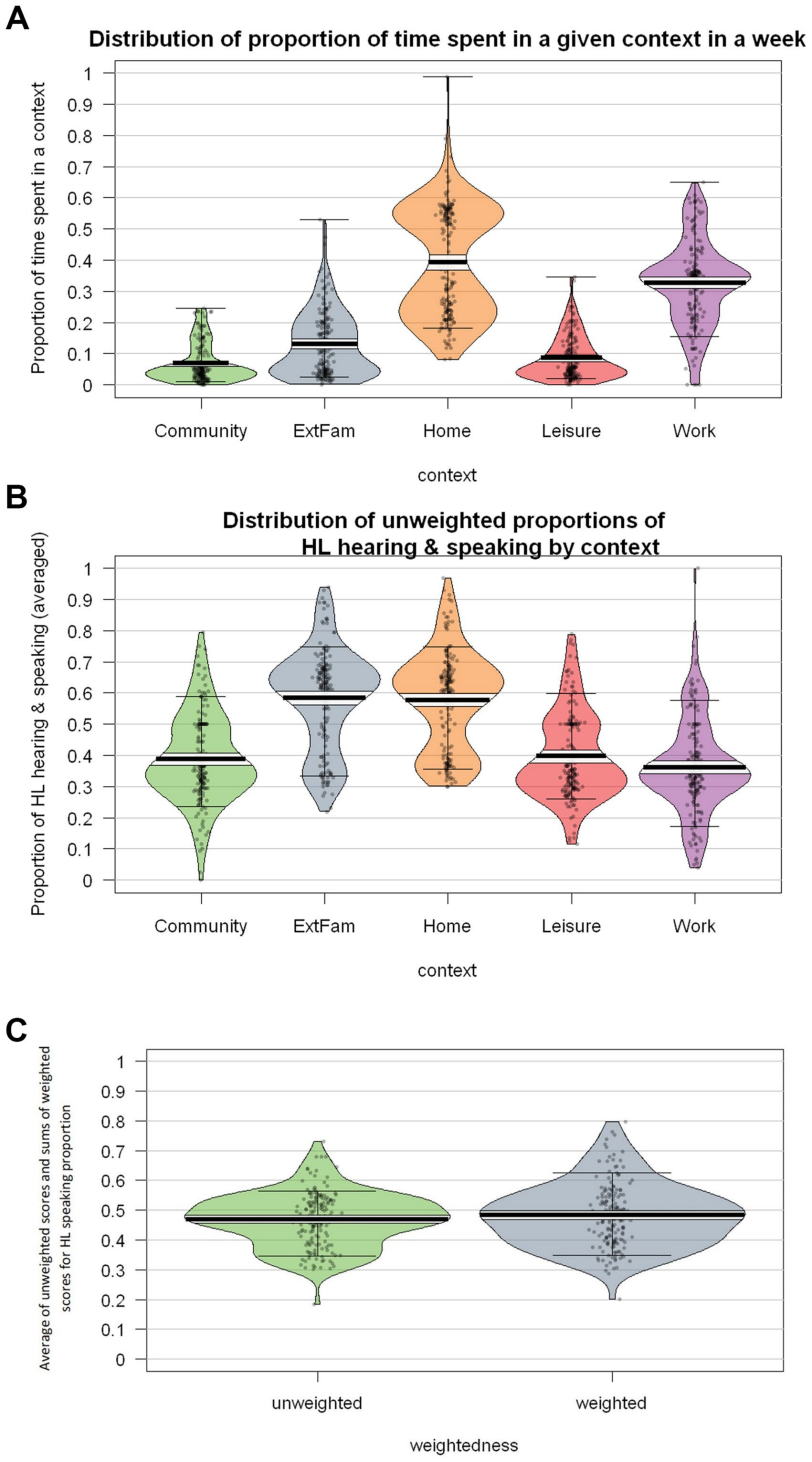
**(A)** Distribution of proportion of time spent in 5 contexts. **(B)** Distribution of unweighted proportion of HL vs. SL hearing and speaking (averaged). **(C)** The distribution of cross-context averages of unweighted HL speaking proportion scores and sums of weighted HL speaking proportion scores.

One of the HeLEx features not available in the LSBQ(-H) is that it documents the estimated amount of time spent in each context. As seen in Figure 10A, this varies substantially both across contexts and within contexts. In general, respondents report spending most time in the Home and Work/School environments.

The proportion of HL use also varies substantially across contexts (highest in the Home and with External Family), as shown in Figure 10B.

When calculating the overall proportion of HL use across contexts, it is important to take into account the actual proportion of the time spent in each context. Figure 10C compares overall proportions with vs. without weighing by time-in-context (based on the calculations explained in the section *Derived measures: Accounting for the actual proportion of time spent in each context*).

The weighted scores seem to be slightly higher than the unweighted scores, likely due to the overlap between the contexts in which participants spend a lot of time in and contexts in which there is a high proportion of HL use, such as Home. The difference did not prove statistically significant (Supplementary Table S9). This will need to be replicated using more diverse groups of Heritage Speakers. In this case, the high proportion of HL use in the work context appears to have balanced out the small amount of time spent with Extended Family.

#### HL input quality and diversity

Two important dimensions of the richness of HL experience are the number and diversity of interlocutors and their level of proficiency in the HL. HeLEx is particularly informative in these respects: it quantifies and “qualifies” HL speakers in each context.

**Figure 11:**
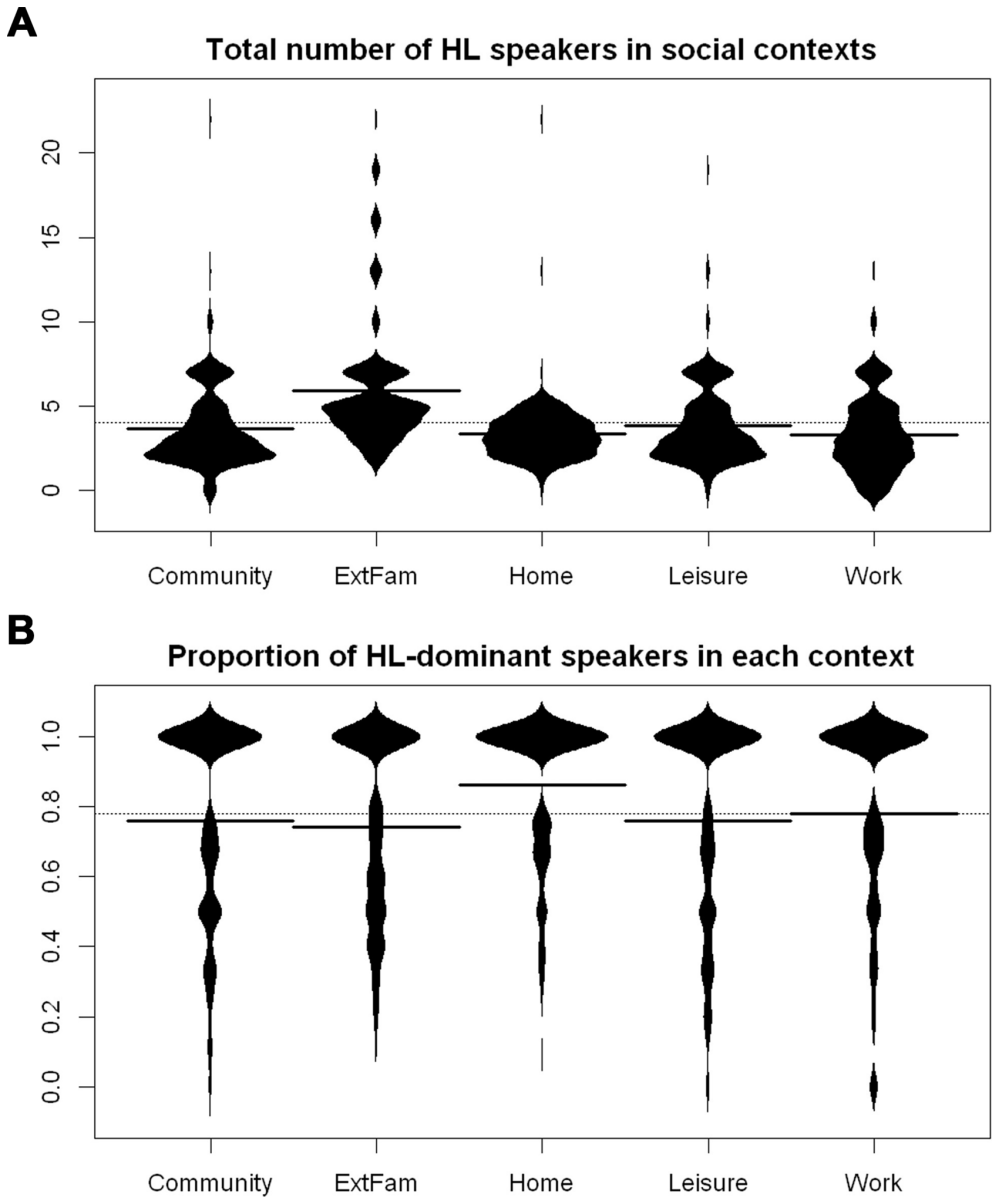
**(A)** Total number of HL speakers in each context. Full lines represent means by contexts, whereas the dotted line represents the overall mean. The clustering of responses into “knots” is an artifact of quantization (cf., Table 5). **(B)** Proportion of speakers who are dominant in HL in each context. The calculations included a check for a number of speakers dominant in HL higher than the total number of speakers, so these were excluded.

The present participant sample seems to get the most diverse HL input in the Family outside of the home context, i.e., ExtFam, judging by the number of HL speakers they spend time with in the context (Figure 11A).

The proportion of speakers with good HL proficiency and HL-dominant speakers follows a broadly similar distribution pattern across contexts, with expectedly higher estimates for the proportion of speakers with good HL proficiency, so we only present the distribution of proportions of HL-dominant speakers (Figure 11B). Interestingly, though, the external family does not seem to have the highest proportion of HL-dominant or HL-proficient speakers. Rather, such speakers are most represented in the Home context.

The results of a linear regression model (Supplementary Table S10) confirm that Home has the highest proportion of HL-dominant speakers, whereas External Family has the lowest proportion, compared to the mean of means of proportion values at every level of the context variable.

## Discussion

In the present study, we analyzed the language experience data from 174 Heritage Speakers of Turkish living in Germany using both a slightly extended version of LSBQ, and HeLEx (“Heritage Language Experience questionnaire”: a new questionnaire amalgamating, modifying, and building on LSBQ and other questionnaires, e.g., the Bilingual Language Profile). We carried out two sets of analyses. The first aimed to ascertain whether the two questionnaires reliably capture the same reality, insofar as the distribution of the resulting measures is sufficiently similar. The second explored the informativity of each questionnaire, in terms of scope and granularity of the derived measures.

Group-level analyses reveal that, despite the distributional differences due to different response scales (see Figures 2, 4), the key variables obtained from each questionnaire are nonetheless sufficiently similar, in that no statistically significant difference was detected in linear regression models probing questionnaire effect on the scaled variables of interest. This was shown in turn for language experience by modality (speaking, listening, reading, writing) and by context (Home, Work or School, Leisure, Community), self-estimated proficiency, experience-based dominance, proficiency-based dominance, and language entropy. The only between-questionnaire difference observed was that LSBQ-H estimates of HL experience across social contexts are significantly lower than the HeLEx ones, especially in the Work/School context. We conclude that the two questionnaires are overall similarly successful at detecting the important distributional patterns in the data.

In terms of informativity, our analyses brought to light several issues regarding response scales (e.g., scales with 4 vs. 7 options, numerical vs. qualitative labels) and response mechanisms (e.g., sliders vs. buttons), which will need to be taken into account in further developments of these and other language experience questionnaires.

First, the minimum and maximum values allocated by design to response scales documenting language experience (i.e., exposure and use) need to take into account the fact that equivalents to 0% (e.g., “never”) and 100% (e.g., “all the time”) will mostly not apply to bilinguals, as even the most dominant ones will still experience their weaker language to some extent. In LSBQ-H, what was by design a 5-point scale effectively turned out to be a 3-point scale as the extremes did not apply. The implication for future questionnaires is that, if unrealistic absolute values are used, the granularity of the scale needs to be adapted accordingly to allow the desired level of detail.

Second, the choice of whether to assign qualitative labels to points on a Likert scale needs careful consideration. Recall that to capture self-reported proficiency, the LSBQ-H employed an 11-point numeric scale with qualitative labels attached to the extreme ends (0, 10) only. In contrast, HeLEx used a 4-point scale with qualitative labels for each of the points (Supplementary Table S5). Our comparative analysis of self-reported proficiency data across questionnaires reveals that the use of qualitative labels such as “pretty well” and “very well” for the top half of a 4-point scale (in HeLEx) returned a less positively skewed distribution than an 11-point numeric scale with qualitative labels attached to the extreme ends (0, 10) only (in the LSBQ-H). Note however that the positive skew of LSBQ-H proficiency responses could have partly stemmed from the response mechanism, sliders, whose potential impact is discussed below. Numeric scales are not necessarily more objective, however: one respondent’s “9” could effectively equate to another’s “7.” The psychometric literature calls for caution in the choice of response scales. Following Dillman et al. (2014), in HeLEx we limited the number of categories on the scale, used symmetrical categories at each end of the scale, and labeled the categories verbally rather than numerically. We believe this led to more consistency across respondents, as it reduces the possibility of different interpretations of what a numerical score of 3 or 7 means in terms of proficiency. A comparison of the scores (derived from each questionnaire) with an objective measure of language proficiency will be needed to settle the issue, but it is beyond the scope of the present paper.

Third, the granularity of ordinal data obtained from Likert scales combined with population characteristics has an impact on the distribution of variables derived from these ordinal data. In this highly balanced population sample, ratio-based dominance-by-proficiency scores (dividing HL proficiency by SL proficiency) featured less variance than difference-based scores (subtracting SL proficiency from HL proficiency; see Figure 6). Further research will need to investigate the informativity of each type of measure when used as predictor variables.

Fourth, the response elicitation mechanism seems to play a role in the response distribution. Slider scales in both questionnaires (to measure proficiency in the LSBQ-H and the proportion of HL use in social contexts in HeLEx) seem to amplify intuitive, categorical choices, where the beginning, mid-point, and end of the slider scale seem to be “hot-spots,” depending on whether the participant considers themselves to be balanced, HL-dominant, or SL-dominant in their language use. This is likely due to the motoric nature of filling in the slider scales, as well as visual presentation. Filling out proficiency scales has different motoric requirements on paper (LSBQ) vs. online (LSBQ-H), and compared to selecting a button response or an option from a menu. On paper, the proficiency scale is filled out by placing a mark on the scale, a movement considered and planned in advance. Its most obvious equivalent in the online questionnaire widget selection is the slider scale. Not to confuse participants and to ensure a consistent point of departure for all responses and participants, slider scales in both HeLEx and LSBQ-H included an initially visible slider tip in the middle of the scale (the tip could have also been initially hidden).^9^ Participants could similarly just click on the desired point on the scale, and the slider tip would appear there. Nevertheless, most participants are likely to have clicked on the tip and dragged it to the desired position. This movement might and more likely to be executed by pulling the slider all the way to the movement limit (beginning or the end point, depending on the participant’s experience), and adjusted slightly from there, or simply leaving it in the middle in case they believe this is the appropriate choice. The “slipperiness” of sliders when choosing a response close to the end of scales could thus cause exponential distribution with a concentration of responses at the end or the beginning of the scale, whereas the inertness of the slider tip when the participant feels a balanced 0.5 response is in order could cause overestimation of balanced scores. In a maximally representative sample of HSs, the slider effect would likely manifest as a trimodal distribution. With the increased necessity for online data collection and translating questionnaires from paper to online platforms, it is important to consider whether the “obvious” online equivalents to paper question formats, e.g., slider scales, are indeed filled out in the same way. As results suggest, this difference in motoric execution of filling in responses is not negligible and could affect the results, in addition to factors such as level of measure derivation or number of response options.

Finally, the treatment of non-responses is not a trivial issue. It is important to distinguish between meaningful non-responses (implying the question does not apply to the participant, or the probed quantity is 0) and non-meaningful non-responses (due to fatigue or non-willingness to respond). There were interactions between the manner of elicitation/calculation of HL use proportion and context, such that slider-derived estimates for HL use proportion for Work/School, Community, and Leisure were significantly lower than hour-derived estimates. The hour-derived estimates particularly for Work/School, Community, and Leisure could have been artificially inflated by excluding quantity non-responses which should have been treated as zeros, since the language experience in question might not have been relevant to some participants. For example, the number of days a participant spends with HL speakers in the Work/School context could have been 0, but the participant left the question on “select” instead of choosing 0 from the menu, which was quantized as NaN and thus technically excluded from plots and statistical models. If these non-responses were turned to zeros to imply that there is no HL use in these contexts, the mean HL use proportions for these contexts would decrease, bringing them closer to slider estimates. We conclude that triangulation or probing similar constructs with several questions of different format and formulation, is highly useful for overcoming such difficulties in interpretation.

While it is not yet established as a standard predictor in bilingualism research, we decided to include Language Entropy in the set of derived variables of interest, as we believe this measure provides an objective estimate of linguistic diversity by context of language experience (Gullifer and Titone, 2020). It might be a reliable proxy for the level of (between-speaker) language mixing (though we leave this for future research). The consistent use of the same five contexts throughout the HeLEx questionnaire facilitates entropy calculations, whereas we show that it is more complex and error-prone to group speakers and derive contexts from various questions in the LSBQ.

We believe the HeLEx questionnaire has a number of advantages as a tool documenting language experience in adult Heritage Speakers. First, language experience questions are all asked in relation to the same set of 5 contexts. This avoids having to reconstruct contexts from by-interlocutor data and avoids having to make assumptions about who the key interlocutors might be in each context (e.g., composition of the homes of young adults). It allows the straightforward combination of information about each context from different questions (e.g., in order to adjust by-context quantities for the actual amount of time spent in each context). We assume that maintaining the same contexts as frame of reference across questions helped reduce the cognitive burden of the questionnaire. Independent evidence would however be required to ascertain that this was the case. Second, we followed the recommendations from the psychometric literature (Dillman et al., 2014, 2016) by systematically using qualitative labels on Likert scales, and by relying on natural metrics (e.g., number of people, hours, days) instead of more ambiguous adverbs of quantification.

One limitation of the study was that the test–retest reliability was not estimated for HeLEx prior to the comparison with LSBQ-H, as we were presented with a unique opportunity to compare HeLEx against LSBQ before completing this step, with a large accessible sample who had recently completed LSBQ-H. Test–retest reliability and (confirmatory) factor analysis should be conducted. Another consequence of “inheriting” data, from a study which did not ensure diversification or representativity across HL populations, was the relatively high homogeneity of the participant sample in terms of language experience and proficiency in both the HL and the SL language. Also, one possible limitation is the time elapsed between the completion of the two questionnaires: several months to a year, leading to potential changes in language experience. However, we believe this is not a cause for concern, as the group-level comparisons reveal a consistent picture.

The current paper suggests that HeLEx is successful in capturing the same constructs as previous questionnaires, namely LSBQ(-H), and provides additional above-mentioned affordances. The findings underline the importance of careful consideration of methodological choices regarding individual difference data elicitation and derivation, and their potential impact on subsequent analyses. The next step of our research is to use HeLEx to document language experience in a highly diverse population of heritage language speakers, and to identify the key language experience variables that predict individual differences in language outcomes (both in terms of language processing and language proficiency).

## Data availability statement

The datasets presented in this study can be found in online repositories. The names of the repository/repositories and accession number(s) can be found at: https://osf.io/mkjax/.

## Ethics statement

The studies involving human participants were reviewed and approved by NSD - Norsk AS Dataforskningssenter (personverntjenester@nsd.no). The patients/participants provided their written informed consent to participate in this study. Written informed consent was obtained from the individual(s) for the publication of any potentially identifiable images or data included in this article.

## Author contributions

CDC and AT co-designed HeLEx, with CDC taking the conceptual and methodological lead. AT implemented the new questionnaire online, created scripts for response quantization and variable derivation, conducted statistical analysis and interpretation, and wrote the first draft of methods, results, and discussion, with assistance and guidance from CDC. CDC co-wrote and edited the manuscript. YR consulted on questionnaire design, wrote introduction, and edited the manuscript. FB consulted on the design of HeLEx, recruited participants, collected the data, and participated in writing. All authors contributed to the article and approved the submitted version.

## Funding

This study was funded by the UiT The Arctic University of Norway Aurora Center for Language Acquisition, Variation & Attrition: The Dynamic Nature of Languages in the Mind (project code 2062165). The publication charges for this article have been funded by a grant from the publication fund of UiT The Arctic University of Norway. This project also received funding from the European Union’s Horizon 2020 research and innovation programme under the Marie Skłodowska-Curie grant agreement No. 799652.

## Conflict of interest

The authors declare that the research was conducted in the absence of any commercial or financial relationships that could be construed as a potential conflict of interest.

## Publisher’s note

All claims expressed in this article are solely those of the authors and do not necessarily represent those of their affiliated organizations, or those of the publisher, the editors and the reviewers. Any product that may be evaluated in this article, or claim that may be made by its manufacturer, is not guaranteed or endorsed by the publisher.

## Supplementary material

The Supplementary material for this article can be found online at: https://www.frontiersin.org/articles/10.3389/fpsyg.2023.1131374/full#supplementary-material

## Footnotes

^1^LSBQ-H also narrows the initial subsection on health to language disorders only, as the rest, e.g., medicine currently being taken and handedness, is relevant for screening EEG participants.

^2^Square brackets [] are used as placeholders in this text. They had been replaced with the appropriate language names in the implemented questionnaire versions.

^3^The questionnaire consists of referenced fields created in the Gorilla task builder to which actual question text is fed through a simple Excel or .csv sheet, which in turn can be easily translated and fed back into the fields to create a different language version.

^4^https://app.gorilla.sc/openmaterials/605087

^5^This assumes that the speakers of HL and SL are separate speakers and not necessarily bilinguals.

^6^We do recognize there is some overlap between the Local Community and Leisure contexts, yet Leisure presupposes more involved relationships, whereas Local Community refers to more brief, everyday, surface engagement.

^7^We also derived dominance score by subtracting SL responses from HL responses and dividing them by the sum of HL and SL responses. This way of calculating dominance gives a similar distribution as do ratios, so we do not include it here.

^8^In a more typically researched heritage speaker population, spending time with HL speakers could likely mean only being exposed to, i.e., hearing, HL. Nevertheless, the proficiency and use patterns from the questions on modalities suggest that these speakers are as likely to speak as they are to hear HL when spending time with other HL speakers.

^9^The LSBQ paper version also has a salient mark and a label (5) at the mid-point of the scale, which could also potentially draw more balanced responses.
