# Supplementary material for: Documenting heritage language experience using questionnaires
Source: Front Psychol. 2023 May 23;14:1131374. doi: 10.3389/fpsyg.2023.1131374 (PMC10243138; doi:10.3389/fpsyg.2023.1131374)
Supplement: Supplementary file 1 [file Data_Sheet_1.docx]

*Table S1: Coefficients of a linear mixed effects model testing modality, questionnaire, and their interactions as predictors for HL experience. HeLEx was the reference level for the Questionnaire variable.*

|  | **freq_of_use_sc** | | |
| --- | --- | --- | --- |
| *Predictors* | *Estimates* | *CI* | *p* |
| (Intercept) | 0.99 | 0.97 – 1.00 | **<0.001** |
| questionnaire [lsbq] | -0.00 | -0.02 – 0.01 | 0.616 |
| Modality 1: Reading | -0.00 | -0.02 – 0.01 | 0.668 |
| Modality 2: Speaking | 0.01 | -0.01 – 0.03 | 0.167 |
| Modality 3: Understanding | 0.01 | -0.01 – 0.03 | 0.365 |
| questionnaire [lsbq] * Modality 1: Reading | -0.00 | -0.03 – 0.02 | 0.801 |
| questionnaire [lsbq] * Modality 2: Speaking | 0.01 | -0.02 – 0.03 | 0.682 |
| questionnaire [lsbq] * Modality 3: Understanding | -0.01 | -0.04 – 0.02 | 0.522 |
| **Random Effects** | | | |
| σ^2^ | 0.02 | | |
| τ_00_ _Participant.Public.ID_ | 0.01 | | |
| ICC | 0.25 | | |
| N _Participant.Public.ID_ | 174 | | |
| Observations | 1392 | | |
| Marginal R^2^ / Conditional R^2^ | 0.005 / 0.251 | | |

*Table S2: Coefficients of a linear mixed effects model testing context, questionnaire, and their interactions as predictors for HL use proportion. HeLEx was the reference level for the Questionnaire variable.*

|  | **HL_props** | | |
| --- | --- | --- | --- |
| *Predictors* | *Estimates* | *CI* | *p* |
| (Intercept) | 0.44 | 0.43 – 0.45 | **<0.001** |
| questionnaire [lsbq] | -0.02 | -0.04 – -0.01 | **0.002** |
| context [1] – Community | -0.04 | -0.06 – -0.02 | **<0.001** |
| context [2] – Home | 0.14 | 0.13 – 0.16 | **<0.001** |
| context [3] – Leisure | -0.04 | -0.05 – -0.02 | **<0.001** |
| questionnaire [lsbq] * context [1] – Community | 0.01 | -0.02 – 0.03 | 0.634 |
| questionnaire [lsbq] * context [2] – Home | 0.01 | -0.02 – 0.03 | 0.570 |
| questionnaire [lsbq] * context [3] – Leisure | 0.02 | -0.01 – 0.04 | 0.243 |
| Observations | 1391 | | |
| R^2^ / R^2^ adjusted | 0.288 / 0.285 | | |

*Table S3: Coefficients of a linear mixed effects model testing modality, questionnaire, and their interactions as predictors for HL proficiency scores.*

|  | **proficiency_sc** | | |
| --- | --- | --- | --- |
| *Predictors* | *Estimates* | *CI* | *p* |
| (Intercept) | 0.99 | 0.98 – 1.00 | **<0.001** |
| questionnaire [lsbq] | 0.01 | -0.00 – 0.02 | 0.150 |
| Modality [1] – Reading | -0.01 | -0.03 – 0.00 | 0.123 |
| Modality [2] - Speaking | 0.02 | 0.00 – 0.03 | **0.010** |
| Modality [3] - Understanding | 0.01 | -0.00 – 0.03 | 0.073 |
| questionnaire [lsbq] * Modality [1] – Reading | 0.01 | -0.01 – 0.03 | 0.233 |
| questionnaire [lsbq] * Modality [2] - Speaking | -0.02 | -0.04 – 0.00 | 0.095 |
| questionnaire [lsbq] * Modality [3] - Understanding | -0.01 | -0.03 – 0.01 | 0.353 |
| **Random Effects** | | | |
| σ^2^ | 0.01 | | |
| τ_00_ _Participant.Public.ID_ | 0.00 | | |
| ICC | 0.21 | | |
| N _Participant.Public.ID_ | 174 | | |
| Observations | 1390 | | |
| Marginal R^2^ / Conditional R^2^ | 0.010 / 0.215 | | |

*Table S4: Coefficients of a linear regression model predicting Experience-Based Dominance (the HL over SL experience ratios averaged across modalities). Reference level for the Questionnaire variable was HeLEx.*

|  | **Dominance** | | |
| --- | --- | --- | --- |
| *Predictors* | *Estimates* | *CI* | *p* |
| (Intercept) | 0.95 | 0.92 – 0.98 | **<0.001** |
| questionnaire [lsbq] | 0.04 | 0.00 – 0.09 | **0.036** |
| **Random Effects** | | | |
| σ^2^ | 0.04 | | |
| τ_00_ _Participant.Public.ID_ | 0.00 | | |
| N _Participant.Public.ID_ | 174 | | |
| Observations | 347 | | |
| Marginal R^2^ / Conditional R^2^ | 0.013 / NA | | |

*Table S5: Coefficients of a linear regression model for predicting Proficiency-Based Dominance.*

|  | **dominance** | | |
| --- | --- | --- | --- |
| *Predictors* | *Estimates* | *CI* | *p* |
| (Intercept) | 0.99 | 0.98 – 1.01 | **<0.001** |
| questionnaire [lsbq] | 0.00 | -0.02 – 0.03 | 0.904 |
| Observations | 345 | | |
| R^2^ / R^2^ adjusted | 0.000 / -0.003 | | |

*Table S6: Coefficients from a linear regression model testing context, questionnaire, as well as their interactions, as predictors for language entropy.*

|  | **Entropy** | | |
| --- | --- | --- | --- |
| *Predictors* | *Estimates* | *CI* | *P* |
| (Intercept) | 0.90 | 0.89 – 0.91 | **<0.001** |
| questionnaire [lsbq] | -0.00 | -0.02 – 0.01 | 0.904 |
| context [1] – Community | 0.01 | -0.01 – 0.03 | 0.170 |
| context [2] – Home | 0.01 | -0.01 – 0.03 | 0.305 |
| context [3] – Leisure | 0.02 | -0.00 – 0.04 | 0.065 |
| questionnaire [lsbq] * context [1] – Community | -0.01 | -0.03 – 0.02 | 0.586 |
| questionnaire [lsbq] * context [2] – Home | 0.01 | -0.02 – 0.03 | 0.638 |
| questionnaire [lsbq] * context [3] – Leisure | 0.00 | -0.02 – 0.03 | 0.794 |
| Observations | 1391 | | |
| R^2^ / R^2^ adjusted | 0.028 / 0.023 | | |

*Table S7: Coefficients from a linear mixed effects model testing Manner of calculation, context, and their interactions as potential predictors for HL proportion in HeLEx.*

|  | **HL_props_speakhear** | | |
| --- | --- | --- | --- |
| *Predictors* | *Estimates* | *CI* | *p* |
| (Intercept) | 0.51 | 0.49 – 0.52 | **<0.001** |
| context1 – Community | -0.03 | -0.05 – -0.00 | **0.017** |
| context2 – ExtFam | 0.00 | -0.02 – 0.02 | 0.905 |
| context3 – Home | 0.04 | 0.01 – 0.06 | **0.001** |
| context4 – Leisure | 0.01 | -0.02 – 0.03 | 0.613 |
| slider [sliders] | -0.05 | -0.06 – -0.03 | **<0.001** |
| context1 – Community * slider [sliders] | -0.05 | -0.08 – -0.02 | **0.003** |
| context2 – ExtFam * slider [sliders] | 0.12 | 0.09 – 0.15 | **<0.001** |
| context3 – Home * slider [sliders] | 0.08 | 0.05 – 0.11 | **<0.001** |
| context4 – Leisure * slider [sliders] | -0.07 | -0.10 – -0.04 | **<0.001** |
| **Random Effects** | | | |
| σ^2^ | 0.03 | | |
| τ_00_ _Participant.Public.ID_ | 0.00 | | |
| ICC | 0.13 | | |
| N _Participant.Public.ID_ | 174 | | |
| Observations | 1724 | | |
| Marginal R^2^ / Conditional R^2^ | 0.153 / 0.261 | | |

*Table S8: Coefficients from the linear mixed effects model comparing entropy in HeLEx calculated using sliders and using hours spent in each context with speakers of each language. The reference level for the Slider (Hours vs. Sliders) variable was Hours.*

|  | **entropy** | | |
| --- | --- | --- | --- |
| *Predictors* | *Estimates* | *CI* | *p* |
| (Intercept) | 0.86 | 0.84 – 0.88 | **<0.001** |
| context1 – Commmunity | -0.03 | -0.06 – -0.01 | **0.003** |
| context2 – ExtFam | 0.02 | -0.00 – 0.04 | 0.068 |
| context3 – Home | 0.03 | 0.01 – 0.05 | **0.011** |
| context4 – Leisure | 0.02 | 0.00 – 0.05 | **0.036** |
| slider [Sliders_speakhear] | 0.04 | 0.02 – 0.05 | **<0.001** |
| context1 – Community * slider [Sliders_speakhear] | 0.04 | 0.00 – 0.07 | **0.026** |
| context2 – ExtFam * slider [Sliders_speakhear] | -0.02 | -0.05 – 0.01 | 0.254 |
| context3 – Home * slider [Sliders_speakhear] | -0.02 | -0.05 – 0.01 | 0.233 |
| context4 – Leisure * slider [Sliders_speakhear] | -0.01 | -0.04 – 0.02 | 0.570 |
| **Random Effects** | | | |
| σ^2^ | 0.03 | | |
| τ_00_ _Participant.Public.ID_ | 0.01 | | |
| ICC | 0.23 | | |
| N _Participant.Public.ID_ | 174 | | |
| Observations | 1724 | | |
| Marginal R^2^ / Conditional R^2^ | 0.024 / 0.252 | | |

*Table S9: Coefficients of a linear regression model testing weightedness by time spent in the context as the predictor for overall HL proportion use score. The reference level for the Weightedness variable (Weighted or Unweighted) was Weighted.*

|  | **Average_Sum_HL_slider_speak** | | |
| --- | --- | --- | --- |
| *Predictors* | *Estimates* | *CI* | *P* |
| (Intercept) | 0.47 | 0.45 – 0.48 | **<0.001** |
| weightedness [weighted] | 0.01 | -0.01 – 0.04 | 0.169 |
| Observations | 344 | | |
| R^2^ / R^2^ adjusted | 0.006 / 0.003 | | |

*Table S10: Linear regression model testing the context as the predictor of the proportion of HL-dominant speakers.*

|  | **Proportion_HLdominantSpeakers** | | |
| --- | --- | --- | --- |
| *Predictors* | *Estimates* | *CI* | *P* |
| (Intercept) | 0.78 | 0.76 – 0.80 | **<0.001** |
| context [1] – Community | -0.02 | -0.06 – 0.02 | 0.239 |
| context [2] – ExtFam | -0.04 | -0.08 – -0.00 | **0.044** |
| context [3] – Home | 0.08 | 0.04 – 0.12 | **<0.001** |
| context [4] – Leisure | -0.02 | -0.06 – 0.02 | 0.285 |
| Observations | 811 | | |
| R^2^ / R^2^ adjusted | 0.025 / 0.020 | | |
